# BAP1 promotes breast cancer cell proliferation and metastasis by deubiquitinating KLF5

**DOI:** 10.1038/ncomms9471

**Published:** 2015-09-30

**Authors:** Junying Qin, Zhongmei Zhou, Wenlin Chen, Chunyan Wang, Hailin Zhang, Guangzhe Ge, Ming Shao, Dingyun You, Zhixiang Fan, Houjun Xia, Rong Liu, Ceshi Chen

**Affiliations:** 1 Key Laboratory of Animal Models and Human Disease Mechanisms of Chinese Academy of Sciences and Yunnan Province, Kunming Institute of Zoology, Chinese Academy of Sciences, Collaborative Innovation Center for Cancer Medicine, Kunming, Yunnan 650223, China; 2 Graduate School of the Chinese Academy of Sciences, Beijing 100039, China; 3 Department of Breast Surgery, Breast Cancer Clinical Research Center, Cancer Hospital, Kunming Medical University, Kunming, Yunnan 650031, China; 4 Department of Pathology, First Affiliated Hospital of Kunming Medical University, Kunming, Yunnan 650032, China; 5 Faculty of Life Science and Technology, Kunming University of Science and Technology, Kunming, Yunnan 650500, China; 6 Kunming Medical University, Kunming, Yunnan 650031, China

## Abstract

The transcription factor KLF5 is highly expressed in basal-like breast cancer and promotes breast cancer cell proliferation, survival, migration and tumour growth. Here we show that, in breast cancer cells, KLF5 is stabilized by the deubiquitinase (DUB) BAP1. With a genome-wide siRNA library screen of DUBs, we identify BAP1 as a bona fide KLF5 DUB. BAP1 interacts directly with KLF5 and stabilizes KLF5 via deubiquitination. KLF5 is in the BAP1/HCF-1 complex, and this newly identified complex promotes cell cycle progression partially by inhibiting *p27* gene expression. Furthermore, BAP1 knockdown inhibits tumorigenicity and lung metastasis, which can be rescued partially by ectopic expression of KLF5. Collectively, our findings not only identify BAP1 as the DUB for KLF5, but also reveal a critical mechanism that regulates KLF5 expression in breast cancer. Our findings indicate that BAP1 could be a potential therapeutic target for breast and other cancers.

## Abstract (web summary)

The zinc finger-containing transcription factor KLF5 drives cell proliferation and migration. Here, the authors show that the debuquitinase BAP1 directly stabilizes KLF5, thus promoting basal-like breast cancer cell-cycle progression and metastasis.

## Graphical abstract

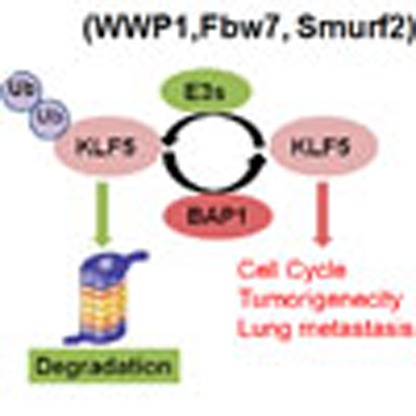

Breast cancer, an increasingly prominent threat confronting women worldwide^1^, is a heterogeneous disease that can be divided, into three major subtypes, by hormone receptor and HER2 expression patterns^2^. Oestrogen receptor (ERα), progesterone receptor and HER2 triple-negative breast cancer (TNBC) comprises ∼15–20% of breast cancers and has the worst prognosis because of a lack of effective therapeutic targets^3^ and distant vital organ metastases^4^. Identification of novel therapeutic targets for TNBC and its metastatic mechanisms is important and needed urgently.

Ubiquitination is a post-translational protein modification that regulates diverse physiological and pathological processes, especially oncogenesis and apoptosis^5^,^6^. The major function of ubiquitination is to target substrate proteins for proteasomal degradation^7^,^8^. Ubiquitination is a reversible process, which is mediated by a large family of deubiquitinating enzymes (DUBs)^9^,^10^. DUBs are well established in regulating cancer development^11^,^12^,^13^. For instance, downregulation of USP9X confers breast cancer resistance to tamoxifen^14^. A20 or CYLD mutations lead to overactivation of the NF-κB pathway in cancers^15^. In recent years, DUBs have become a class of novel anticancer targets^16^,^17^. The small-molecule WP1130 inhibits several DUBs and triggers apoptosis in cancer cells^18^; the identification of novel DUB inhibitors is important for cancer therapy.

*KLF5* (Krüppel-like zinc-finger transcription factor 5) is a transcription factor that is highly expressed in ERα-negative basal subtype breast cancers^19^. High *KLF5* messenger RNA (mRNA) and protein levels have been reported as a potent biomarker for unfavourable prognosis for breast cancer patients^20^,^21^. Furthermore, our previous studies demonstrated that KLF5 promoted cell proliferation, survival and tumour growth, partially through inducing the transcription of downstream target genes, such as fibroblast growth factor-binding protein 1 *(FGF-BP)*^22^,^23^,^24^ and microsomal prostaglandin E2 synthase 1 (*mPGES1)*^25^. These findings collectively define KLF5 as a potent therapeutic target for basal TNBC and other cancers. However, transcription factors are not ideal targets for drug development. Thus, it is important to identify KLF5 upstream positive regulators, which may be better therapeutic targets for cancer treatment. KLF5 has been identified as an unstable protein that is ubiquitinated by WWP1, SCF^Fbw7^ and Smurf2 E3 ligases and degraded^26^,^27^,^28^,^29^,^30^.

We hypothesize that KLF5 DUBs should stabilize the KLF5 protein and possess oncogenic functions in breast cancer. To identify DUBs for KLF5, we screened a short interfering RNA (siRNA) library against 87 human DUBs. BAP1 was identified as a DUB for KLF5. BAP1 promotes breast cancer cell proliferation and migration *in vitro* and tumour growth and lung metastasis *in vivo*. Our results suggest that BAP1 and KLF5 are potential therapeutic targets for breast cancer.

## Results

### Identification of candidate DUBs for KLF5

**Figure 1:**
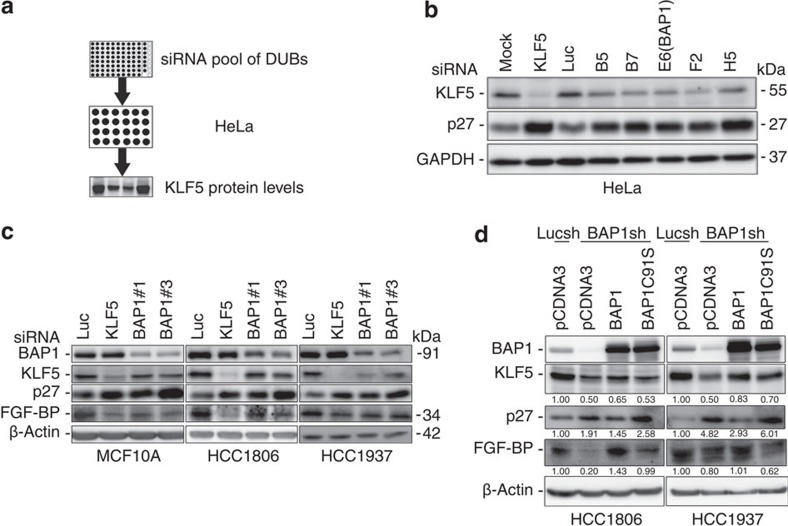
BAP1 is a candidate DUB that maintains KLF5 protein stability. (**a**) Diagram of the screening procedure used to identify KLF5 DUBs. Eighty-seven siRNA pools were transfected into HeLa cells individually. Endogenous KLF5 protein levels were examined by WB after 48 h. (**b**) Validation of the five candidate KLF5 DUBs in HeLa cells. p27 is inhibited by KLF5. The siRNAs were transfected into HeLa cells, respectively, and endogenous KLF5 protein levels were measured by WB after 48 h. (**c**) BAP1 knockdown decreased KLF5 and FGF-BP, but increased p27 protein levels in various breast epithelial cell lines. MCF10A, HCC1806 and HCC1937 cells were transfected with control or two independent BAP1 siRNAs for 48 h. Luciferase (Luc) siRNA was used as the negative control. KLF5 siRNA was used as the positive control. The endogenous KLF5, FGF-BP and p27 protein levels were measured by WB. (**d**) BAP1 stable knockdown by shRNA#6 induced KLF5, FGF-BP and p27 protein expression changes were rescued by transient overexpression of BAP1, but not BAP1-C91S in HCC1806 and HCC1937 cells. For more data see Supplementary Fig. 10.

To identify potential DUBs for KLF5, we screened a siRNA library consisting of siRNA pools against 87 human DUBs using the procedure shown in Fig. 1a. We knocked down individual human DUBs using pooled siRNAs (a mixture of three siRNAs per gene) in HeLa cells (Fig. 1a) for 2 days and detected the KLF5 protein levels by western blotting (WB). The quantification results for KLF5 protein levels are shown in Supplementary Fig. 1A. Twenty-seven candidate DUB genes were subjected to the second round of screening. In the end, five candidates were confirmed in both HeLa (Fig. 1b) and MCF10A cells (Supplementary Fig. 1B), because their knockdown obviously downregulated the KLF5 protein levels. Protein levels of p27, a KLF5 inhibited target gene (see details below), were also upregulated by the knockdown of these DUBs (Fig. 1b; Supplementary Fig. 1B). Among the candidate genes, BAP1 (BRCA1-associated protein 1) drew our special attention because it has been reported to localize in the nucleus and promote breast cancer cell growth similar to KLF5 (refs 31, 32, 33). In addition, BAP1 is frequently mutated in mesothelioma, uveal melanoma, melanocytic tumour, renal cell carcinoma and other cancers^31^,^34^. So far, the function and mechanism of BAP1 are still not clear.

BAP1 is a member of the ubiquitin C-terminal hydrolase DUB subfamily, which was originally discovered as a novel DUB interacting with BRCA1 (ref. 35). Recently, a flurry of genetic studies has reported that BAP1 could be a tumour suppressor gene inactivated in various cancers^36^, such as mesothelioma^37^,^38^, uveal melanomas^39^, melanocytic tumours^40^ and renal cell carcinoma^41^. BAP1 has been reported to suppress cell growth through its catalytic activity and nuclear localization^31^,^42^. Moreover, RNA-mediated depletion of BAP1 promoted cell cycle G1–S progression^43^. In sharp contrast, RNA interference-mediated depletion of BAP1-induced cell cycle progression defects and inhibited cell proliferation *in vitro*^33^,^44^, suggesting that BAP1 may have dual roles in cancer development. Therefore, it is urgent to validate the function of BAP1 in breast cancer *in vivo* and characterize its functional mechanisms.

To further confirm whether BAP1 increases KLF5 protein stability, we transfected two different siRNAs targeting different regions of BAP1 into MCF10A and breast cancer cell lines (HCC1806 and HCC1937). BAP1 knockdown decreased the endogenous protein levels of KLF5 and its downstream target gene *FGF-BP*^23^ (Fig. 1c). Knockdown of either KLF5 or BAP1 upregulated p27 protein levels in these breast cell lines as well (Fig. 1c; Supplementary Fig. 1C). Similar results were observed in another TNBC cell line SUM149PT (Supplementary Fig. 1C).

To eliminate off-target effects of BAP1 siRNAs, we re-expressed wild-type (WT) BAP1 and inactive mutant BAP1-C91S in BAP1 stable knockdown HCC1806 and HCC1937 cells (Fig. 1d; Supplementary Fig. 10). WT BAP1, but not mutant BAP1-C91S, obviously elevated the KLF5 and FGF-BP protein levels and inhibited the p27 protein levels in both cell lines (Fig. 1d).

Next, we tested whether BAP1 regulates the *KLF5* mRNA expression levels. The *KLF5* mRNA levels were not decreased by BAP1 knockdown in the MCF10A and HCC1806 cells (Supplementary Fig. 1D). Thus, BAP1 maintains KLF5 protein expression. Taken together, BAP1 is a strong candidate DUB for KLF5 because it stabilizes the KLF5 protein in breast cells.

### BAP1 stabilizes the KLF5 protein through deubiquitination

**Figure 2:**
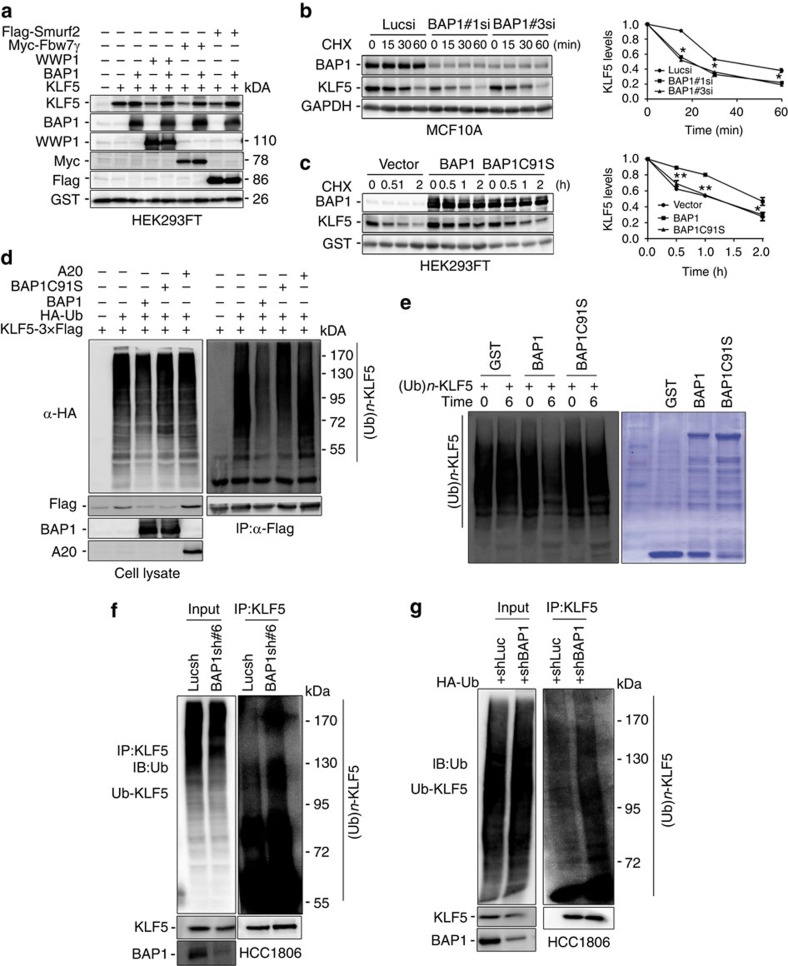
BAP1 stabilizes and deubiquitinates KLF5. (**a**) BAP1 antagonized E3 ligase-mediated degradation of KLF5. HEK293FT cells were transfected with the BAP1 or KLF5 E3 ligases (WWP1, Fbw7γ and Smurf2) for 48 h. The cell lysates were analysed by WB. (**b**) BAP1 knockdown enhanced KLF5 protein degradation. MCF10A cells were transfected with Luc or varying BAP1 siRNAs. After treating the cells with cycloheximide (CHX) for an indicated time, the expression of endogenous KLF5 protein was analysed by WB (left panel). The band intensity of KLF5 for each time point was quantified by ImageJ and plotted (right panel). Experiments were repeated for three times, and a representative experiment is presented. Error bars represent s.d. Every experimental group was compared with the control Lucsi group, *P<0.05, *t*-test. (**c**) BAP1 overexpression stabilized the KLF5 protein. KLF5 was co-expressed with BAP1 or BAP1-C91S in HEK293FT cells. After the cells were treated with CHX, KLF5 protein levels were analysed by WB (left panel). GST was used as transfection and loading controls. Quantitative data are shown in the right panel. Experiments were repeated for three times, and a representative experiment is presented. Error bars represent s.d. Every experimental group was compared with the vector group, *P<0.05; **P<0.01, *t*-test. (**d**) BAP1 decreased KLF5 ubiquitination in HEK293FT cells. KLF5-3 × Flag and HA-Ub were co-expressed with BAP1, BAP1-C91S or A20. After the cells were treated with MG132 for 6 h, KLF5 proteins were immunoprecipitated and the polyubiquitinated KLF5 proteins were detected by WB using an anti-HA antibody. (**e**) BAP1 deubiquitinates KLF5 *in vitro*. Ubiquitinated KLF5 was purified from MG132-treated HEK293FT cells and then incubated with purified GST-tagged BAP1 or BAP1-C91S *in vitro*. The polyubiquitinated KLF5 proteins were examined by WB. (**f**) Stable BAP1 knockdown HCC1806 cells and Lucsh cells were treated with MG132 (20 μM) for 6 h. Cell lysates were immunoprecipitated with the anti-KLF5 antibody and KLF5 ubiquitination was examined by WB using the anti-Ub antibody. (**g**) The HA-Ub plasmid was transiently transfected into Lucsh and BAP1sh HCC1806 cells for 48 h. The cells were treated with MG132 (20 μM) for 6 h. Extracts were immunoprecipitated with KLF5 antibody, and KLF5 ubiquitination was examined by WB using the anti-Ub antibody.

The KLF5 protein is ubiquitinated by at least three E3 ligases, including WWP1, SCF^Fbw7^ and Smurf2. To test whether BAP1 can antagonize E3 ligase-mediated KLF5 degradation, we transfected KLF5 E3 ligases and BAP1 into HEK293FT cells and detected the KLF5 protein levels. As expected, the three E3 ligases, including WWP1, Fbw7 and Smurf2, downregulated the exogenous KLF5 protein levels, and BAP1 overexpression blocked KLF5 protein degradation induced by each of the E3 ligases (Fig. 2a). Similar results were obtained in HCC1806 breast cancer cells (Supplementary Fig. 1E). To further investigate whether endogenous BAP1 protects KLF5 protein from degradation, we knocked down BAP1 using two different siRNAs in MCF10A cells and measured the KLF5 protein half-lives with the cycloheximide chase assay. As expected, when BAP1 was depleted, the KLF5 protein half-life was decreased from 40 to 23 min (Fig. 2b). In addition, we measured the exogenous KLF5 protein half-lives after we overexpressed WT BAP1 and BAP1-C91S in HEK293FT cells. The KLF5 protein half-life was extended from 60 to 120 min by WT BAP1, but not by BAP1-C91S (Fig. 2c).

To directly test whether BAP1 regulates KLF5 protein stability dependent on deubiquitination, we transfected KLF5-3 × Flag, haemagglutinin (HA)-Ub and BAP1 or BAP1-C91S into HEK293FT cells and treated the cells with the proteasome inhibitor MG132 to block protein degradation. Polyubiquitinated KLF5 proteins were detected using an anti-HA antibody (Fig. 2d). BAP1, but not BAP1-C91S, markedly decreased KLF5 protein polyubiquitination (Fig. 2d, lanes 3 and 4), whereas another DUB, A20, did not decrease KLF5 polyubiquitination (Fig. 2d, lane 5). Similar results were observed in the HCC1806 breast cancer cell line (Supplementary Fig. 2A). Furthermore, we tested whether purified recombinant BAP1 can directly decrease KLF5 polyubiquitination *in vitro*. Glutathione *S*-transferase (GST)-BAP1 and GST-BAP1-C91S were expressed and purified from an *Escherichia coli* system. Polyubiquitinated KLF5 proteins purified from mammalian cells by immunoprecipitation were incubated with GST, GST-BAP1 or GST-BAP1-C91S. Compared with GST and BAP1-C91S, BAP1 specifically decreased KLF5 polyubiquitination (Fig. 2e; Supplementary Fig. 2B). Since KLF5 is predominately ubiquitinated with K48-linked rather than K63-linked polyubiquitin chains (Supplementary Fig. 2D,E), BAP1 appeared to remove K48-linked polyubiquitin chains from KLF5. Interestingly, BAP1 cannot catalyse free K48-linked polyubiquitin chains *in vitro* (Supplementary Fig. 2C). Finally, we demonstrated that knockdown of endogenous BAP1 increased the ubiquitination of endogenous KLF5 in HCC1806 (Fig. 2f,g). Collectively, these results suggest that BAP1 is a specific DUB for KLF5 and that BAP1 increases KLF5 protein stability in a DUB activity-dependent manner.

### BAP1 interacts directly with KLF5

**Figure 3:**
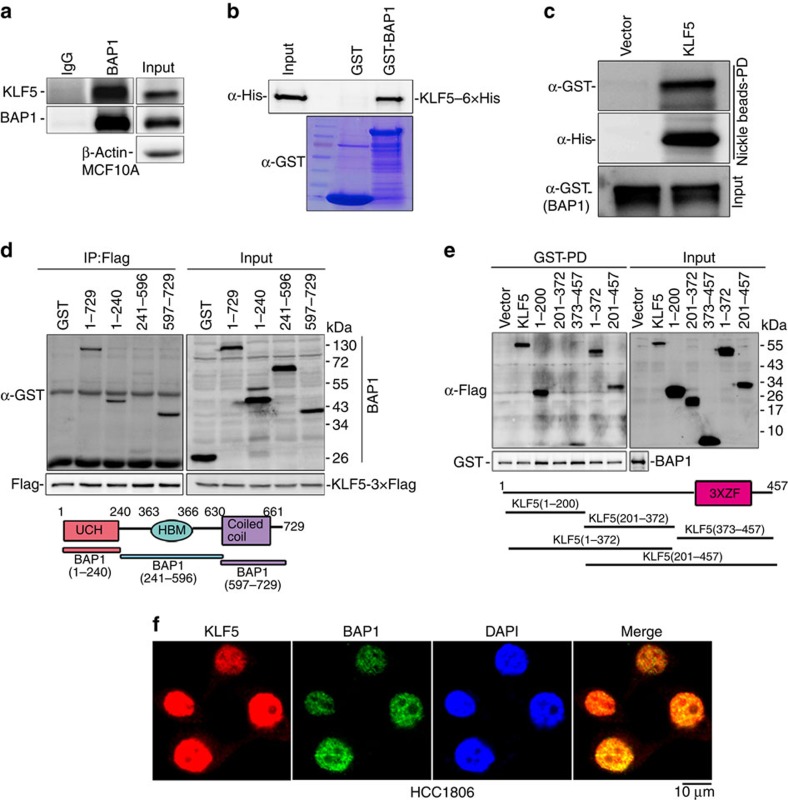
The BAP1 and KLF5 proteins directly interact. (**a**) Endogenous BAP1 and KLF5 proteins interact with one another in MCF10A cells. Endogenous BAP1 proteins were immunoprecipitated with the anti-BAP1 antibody. Immunoglobulin (Ig)G serves as the negative control. Endogenous KLF5 was detected by WB. (**b**) Purified recombinant GST-BAP interacts with KLF5-6 × His. GST-BAP1 and GST proteins were pulled down with glutathione beads. KLF5-6 × His was detected by WB. Purified GST and GST-BAP1 were detected by Coomassie blue staining. (**c**) Purified recombinant KLF5-6 × His interacts with GST-BAP. KLF5-6 × His proteins were pulled down with nickel beads. GST-BAP was detected by WB. (**d**) Mapping the domains of BAP1 that interact with KLF5. GST-fused full-length or deletion constructs of BAP1 (a schematic diagram is shown below the panel) were co-expressed with KLF5-3 × Flag in HEK293FT cells. KLF5-3 × Flag proteins were immunoprecipitated with Flag-M2 beads, and BAP1 proteins were detected by WB. (**e**) Mapping the domains of KLF5 that interact with BAP1. Flag-tagged full-length or deletion constructs of KLF5 (a schematic diagram is shown below the panel) and GST-BAP1 were transfected into HEK293FT cells. GST-BAP1 proteins were pulled down with glutathione beads, and KLF5 proteins were detected by WB. (**f**) BAP1 and KLF5 are co-localized in the nucleus of HCC1806 cells. The cellular location of KLF5 and BAP1 was examined by immunofluorescence staining. DAPI was used to stain the DNA. Scale bar, 10 μM. DAPI, 4,6-diamidino-2-phenylindole.

As a KLF5 DUB, BAP1 and KLF5 proteins should interact with one another. To examine this, KLF5-3 × Flag and BAP1 were co-transfected into HEK293FT cells for protein–protein interaction analysis by co-immunoprecipitation. When KLF5-3 × Flag was immunoprecipitated with an anti-Flag antibody, BAP1 was also co-immunoprecipitated (Supplementary Fig. 3A). Next, we demonstrated that endogenous BAP1 and KLF5 proteins also interacted with one another in MCF10A cells (Fig. 3a). The interaction appeared to be enhanced by serum stimulation in MCF10A (Supplementary Fig. 3B). Furthermore, we expressed and purified recombinant GST-BAP1 and KLF5-6 × His. When purified GST-BAP1 was pulled down by glutathione beads, KLF5-6 × His was detected in the complex (Fig. 3b). Vice versa, when KLF5-6 × His was pulled down by nickel beads, GST-BAP1 was detected in the complex (Fig. 3c). These results suggest that BAP1 and KLF5 directly interact with one another.

BAP1 contains a ubiquitin C-terminal hydrolase domain at its N terminus, an HCF-1 binding motif (HBM) that interacts with HCF-1 and a coiled–coil domain at its C terminus^31^,^33^. To identify which regions of BAP1 are responsible for the KLF5 interaction, we generated a series of GST-fused BAP1 deletion mutants and transfected them into HEK293FT cells with KLF5-3 × Flag. Full-length BAP1 (1–729), the BAP1 N terminus (1–240) and the BAP1 C terminus (597–729) interacted with KLF5-3 × Flag (Fig. 3d). Only the middle part of BAP1 (241–596), containing the HBM motif, did not interact with KLF5 (Fig. 3d). We also mapped the KLF5 protein regions involved in the interaction with BAP1 by constructing a series of KLF5-3 × Flag-truncated mutants (Fig. 3e). After these mutants were expressed in HEK293FT cells together with GST-BAP1, we pulled down GST-BAP1 with glutathione beads and found that full-length KLF5 (1–457), the KLF5 N terminus (1–200, 1–372) and the KLF5 C terminus (373–457, 201–457) interacted with BAP1. Only the middle part of KLF5 (201–372) did not interact with GST-BAP1 (Fig. 3e). Finally, we confirmed that both endogenous BAP1 and KLF5 are co-localized in the nuclei of HCC1806 and HCC1937 cells using immunofluorescence staining (Fig. 3f; Supplementary Fig. 3C). Taken together, these results suggest that BAP1 directly interacts with KLF5 and that the interaction is mediated by the N- and C termini of BAP1 and KLF5.

### The KLF5/BAP1/HCF-1 complex promotes cell cycle progression

**Figure 4:**
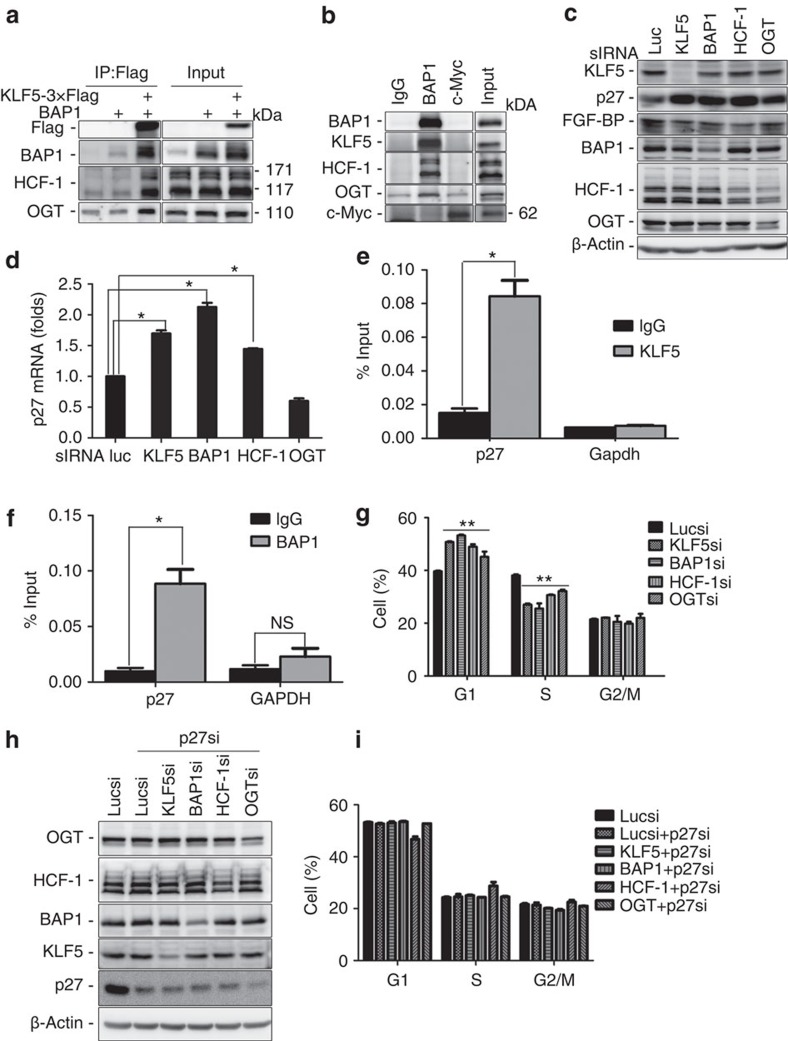
The KLF5/BAP1/HCF-1 complex promotes cell cycle progression through p27. (**a**) Exogenous KLF5 interacted with BAP1, HCF-1 and OGT. KLF5-3 × Flag and BAP1 were co-expressed in HEK293FT cells. When KLF5 was immunoprecipitated with anti-Flag-M2 beads, exogenous BAP1 and endogenous HCF-1 and OGT co-immunoprecipitated. (**b**) Endogenous KLF5, BAP1, HCF-1 and OGT proteins are in the same protein complex. The HCC1806 cell lysates were immunoprecipitated with the anti-BAP1 antibody. Endogenous KLF5, HCF-1 and OGT proteins co-immunoprecipitated. The c-Myc protein was not in the protein complex. Immunoglobulin (Ig)G was used as the negative control. (**c**) Knockdown of each member of the KLF5/BAP1/HCF-1 complex in HCC1806 cells upregulated p27 protein levels and downregulated FGF-BP protein levels. (**d**) Knockdown of KLF5, BAP1 and HCF-1, but not OGT, upregulated *p27* mRNA levels in HCC1806 cells as measured by RT–quantitative (q)PCR (*n*=3). The expression levels of *p27* were normalized to β-actin (mean±s.d. from three experiments in triplicate). Every experimental group (KLF5si, BAP1si, HCF-1si and OGTsi) was compared with the Lucsi group, *P<0.05, *t*-test. (**e**) KLF5 binds to the *p27* gene promoter as determined by ChIP assays in HCC1806 cells. Either IgG- or ChIP-grade anti-KLF5 antibody was used for ChIP. ChIP signals were determined by qPCR and calculated as percentage IP DNA/input DNA (mean±s.d. from three experiments). IgG was served as a negative control. The KLF5 group was compared with the IgG group. *P<0.05, *t*-test. (**f**) BAP1 binds to the *p27* gene promoter as determined by ChIP assays in HCC1806 cells (mean±s.d. from three experiments). *P<0.05; NS, not significant, *t*-test. (**g**) Knockdown of each member of the KLF5/BAP1/HCF-1 complex in HCC1806 by siRNA for 48 h decreased G1/S cell cycle progression (*n*=3). Data were presented as percentage G1, S or G2/M phase (mean±s.d. from three experiments in duplicate). Every experimental group (KLF5si, BAP1si, HCF-1si and OGTsi) was compared with the Lucsi group, **P<0.01, *t*-test. (**h**) Knockdown of p27 in combination with KLF5, BAP1, HCF-1 or OGT knockdown in HCC1806 cells. The protein levels of p27, KLF5, BAP1, HCF-1 and OGT were analysed by WB. (**i**) The HCC1806 cell cycle G1-phase arrest induced by the knockdown of KLF5, BAP1, HCF-1 or OGT was rescued by the depletion of p27 (*n*=3). Data are presented as percentage G1, S or G2/M phase (mean±s.d. from three experiments in duplicate).

BAP1 has been reported to form a protein complex with host cell factor (HCF-1), *O*-linked *N*-acetylglucosaminetransferase (OGT), and several other proteins^9^,^33^,^42^,^44^,^45^,^46^. Because KLF5 interacted with BAP1 directly, we wondered whether KLF5 was in the BAP1/HCF-1 complex. To address this question, KLF5-3 × Flag and BAP1 were co-transfected into HEK293FT cells. When KLF5 was immunoprecipitated, exogenous BAP1 and endogenous HCF-1 and OGT were also co-immunoprecipitated (Fig. 4a). Next, we immunoprecipitated endogenous BAP1 using an anti-BAP1 antibody in HCC1806 cells; when BAP1 was pulled down, endogenous KLF5, HCF-1 and OGT proteins were detected (Fig. 4b). The c-Myc protein was not co-immunoprecipitated and the negative control immunoglobulin G did not immunoprecipitate any of these proteins, suggesting that the interaction is specific and that KLF5 could be a component of the BAP1/HCF-1 protein complex.

Next, we explored the function of the KLF5/BAP1/HCF-1 complex. Given that depletion of BAP1 or KLF5 markedly increased p27 protein levels, we speculated that the protein complex might regulate the expression of p27 and FGF-BP (a KLF5 target gene). As expected, knockdown of BAP1, HCF-1, OGT or KLF5 markedly increased p27 and decreased FGF-BP protein levels in HCC1806 cells (Fig. 4c). We took the *p27* gene as an example and performed quantitative reverse transcription–PCR to measure mRNA levels. Knockdown of KLF5, BAP1 or HCF-1, but not OGT, significantly increased *p27* mRNA levels (Fig. 4d). Finally, chromatin immunoprecipitation (ChIP) assay results confirmed that both KLF5 and BAP1 bound to the *p27* gene promoter (Fig. 4e,f).

The *p27* gene has been well documented to inhibit cell cycle progression. Because the BAP1/KLF5/HCF-1 protein complex inhibited p27 expression, we examined the cell cycle distribution after knocking down KLF5, BAP1, HCF-1 and OGT, respectively, in HCC1806 cells. As expected, the number of G1-phase cells were significantly increased and the number of S-phase cells significantly decreased when KLF5, BAP1, HCF-1 or OGT were knocked down (Fig. 4g). To test whether the KLF5/BAP1/HCF complex promotes cell cycle progression through p27, we knocked down p27 together with KLF5, BAP1, HCF-1 or OGT (Fig. 4h). The G1-phase arrest induced by the knockdown of any component of the KLF5/BAP1/HCF-1 protein complex was rescued by the depletion of p27 (Fig. 4i). These results suggest that the KLF5/BAP1/HCF-1 protein complex promotes cell cycle progression partially through inhibiting *p27* gene transcription.

### BAP1 promotes cell proliferation through KLF5

**Figure 5:**
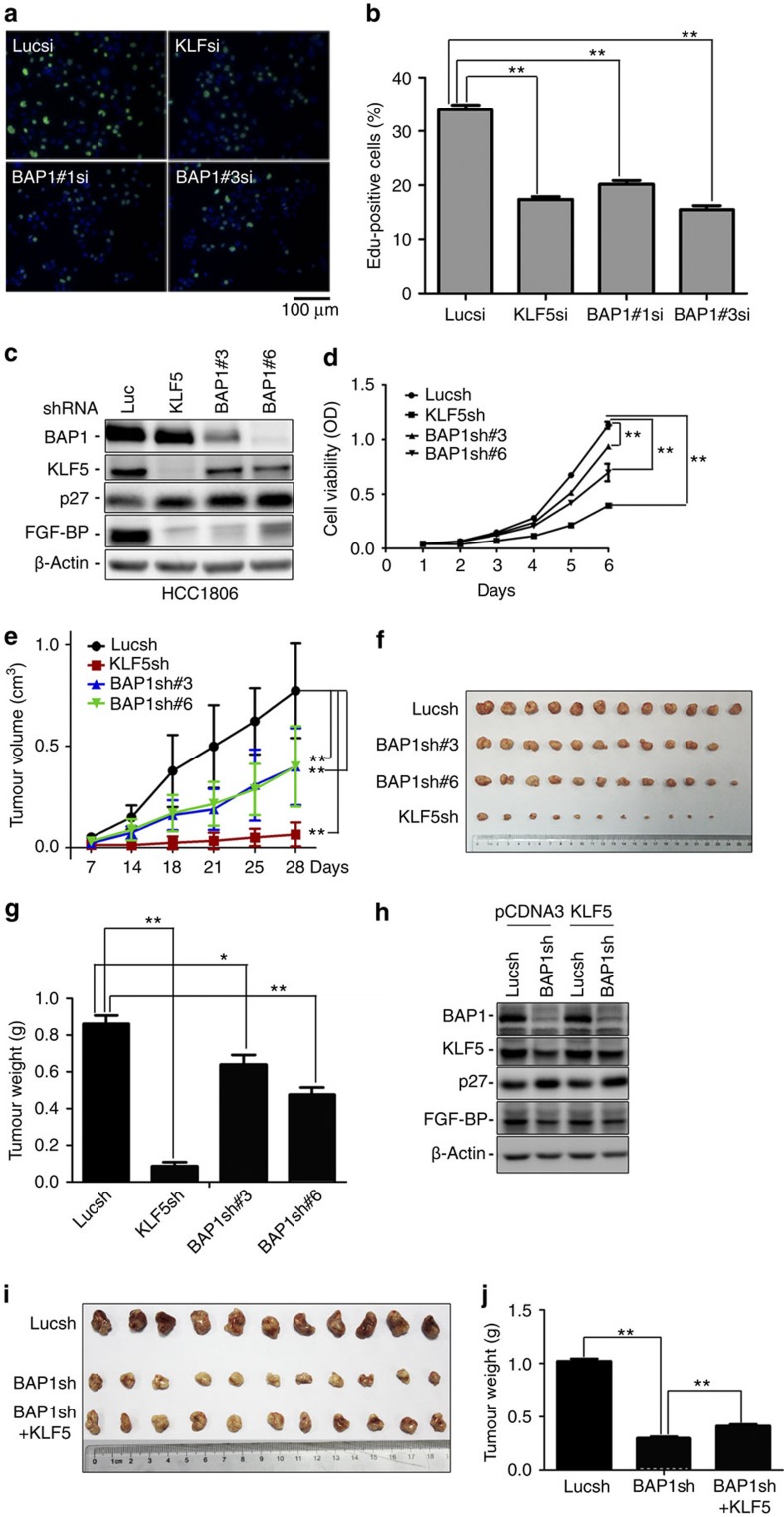
BAP1 knockdown partially suppresses cell proliferation through KLF5. (**a**) Knockdown of either BAP1 or KLF5 significantly decreased DNA synthesis in HCC1806 cells. The green dye represented the proliferative cells and the blue dye represented the total cells. Representative images are shown. Scale bar, 100 μm. (**b**) The quantitative results (*n*=6) of **a**. The percentages represented the proportion of green cells in the total cells. The *t*-test was done on raw percentages without any transformation (mean±s.d. from six photos in two independent experiments). Every experimental group (KLF5si, BAP1#1si and BAP1#3si) was compared with the Lucsi group, **P<0.01, *t*-test. (**c**) Stable knockdown of BAP1 decreased KLF5 and FGF-BP and increased p27 protein levels in HCC1806 cells. (**d**) Stable knockdown of either BAP1 or KLF5 inhibited HCC1806 cell growth *in vitro* as measured by the sulforhodamine B assay (*n*=4). **P<0.01, *t*-test. The cells stably transfected Luc, KLF5, BAP1#3 or BAP1#6 shRNAs were planted into 24 wells and cell viability was measured every day. Data points represent the mean±s.d. of three duplicates per group. Statistical significance was determined by a *t*-test. Every experimental group (KLF5sh, BAP1#3sh and BAP1#6sh) was compared with the Lucsh group. **P<0.01, *t*-test. (**e**) Stable knockdown of either BAP1 or KLF5 suppressed HCC1806 tumour growth in NOD-SCID mice. Xenograft tumour growth was measured twice a week. Data points represent the mean±s.d. of six mice per group (12 tumours). Statistical significance was determined by a *t*-test. Every experimental group (KLF5sh, BAP1#3sh and BAP1#6sh) was compared with the Lucsh group. **P<0.01, *t*-test. (**f**) Stable knockdown of either BAP1 or KLF5 generated smaller xenografts compared with the Lucsh control at day 28. (**g**) Stable knockdown of either BAP1 or KLF5 significantly decreased tumour weight compared with the Lucsh control at day 28 (*n*=12). Error bars represent s.d. *P<0.05 and **P<0.01, *t*-test. (**h**) KLF5 was transiently overexpressed in BAP1 stable knockdown HCC1806 cells. KLF5 protein levels were modestly restored in the BAP1 stable knockdown cells. (**i**) KLF5 transient overexpression modestly increased tumour masses in BAP1 stable knockdown HCC1806 cells (*n*=11). (**j**) KLF5 transient overexpression modestly but significantly increased tumour weight in BAP1 stable knockdown HCC1806 cells (*n*=11). Error bars represent s.d. **P<0.01, *t*-test.

Our previous studies demonstrated that KLF5 promoted breast cancer cell proliferation and tumorigenesis^22^,^23^. However, the function of BAP1 in cancer is controversial. We knocked down BAP1 in HCC1806 cells using two different siRNAs and examined DNA synthesis. Consistent with the cell cycle analysis, depletion of either BAP1 or KLF5 significantly inhibited DNA synthesis (Fig. 5a,b). Similar results were obtained in HCC1937 and MDA-MB-468 TNBC cell lines (Supplementary Fig. 4A,B). Interestingly, depletion of either BAP1 or KLF5 did not significantly inhibited DNA synthesis in MDA-MB-231, which is a KLF5-negative breast cancer cell line (Supplementary Fig. 4C). Next, we generated BAP1 and KLF5 stable knockdown HCC1806 cell lines. Consistent with transient knockdown results, p27 protein levels were upregulated and FGF-BP protein levels were downregulated (Fig. 5c). Cell growth was suppressed as measured by the sulforhodamine B (SRB) assay when either BAP1 or KLF5 was silenced (Fig. 5d). Similar results were obtained in HCC1937 and MDA-MB-468 cell lines (Supplementary Fig. 4A,B). Again, depletion of either BAP1 or KLF5 did not significantly decreased MDA-MB-231 cell growth (Supplementary Fig. 4C). These results indicate that BAP1 promotes KLF5-positive breast cell proliferation *in vitro*.

Next, we stably overexpressed BAP1 in HCC1937 and measured cell growth. As shown in Supplementary Fig. 5A,B, BAP1 overexpression elevated the KLF5 protein level, decreased the p27 protein level and significantly promoted cell growth. When KLF5 is depleted, BAP1 failed to promote cell growth (Supplementary Fig. 5C,D). These results confirmed that BAP1 promotes cell proliferation *in vitro* through KLF5 in part.

We next validated the function of BAP1 *in vivo*. BAP1- and KLF5-depleted HCC1806 cells (5 × 10^6^ per spot, *n*=6) were subcutaneously injected into nonobes ediabetic severe combined immunodeficient (NOD-SCID) mice. During a 1-month period, BAP1 and KLF5 knockdown cancer cells grew significantly slower than the Lucsh control cells (Fig. 5e,f). Two different BAP1 shRNAs (#3 and #6) showed similar results. The average tumour weights of BAP1sh#3, BAP1sh#6 and KLF5sh xenografts were significantly less than that of the Lucsh xenografts at day 28 (Fig. 5g). It is clear that KLF5 knockdown inhibits tumour growth much more than BAP1 knockdown in this model (Fig. 5e–g). Finally, we tested whether BAP1 promoted HCC1806 tumour growth through KLF5. Because KLF5 was downregulated in the BAP1 knockdown cells (Fig. 5c,h), we transiently overexpressed KLF5 in the BAP1sh and Lucsh cells (Fig. 5h). Transient overexpression of KLF5 partially but significantly rescued the BAP1 knockdown-induced tumour growth suppression (Fig. 5i,j). Taken together, these results demonstrate that BAP1 promotes HCC1806 breast cancer tumorigenesis partially by stabilizing KLF5.

### BAP1 and KLF5 promote breast cancer lung metastasis

**Figure 6:**
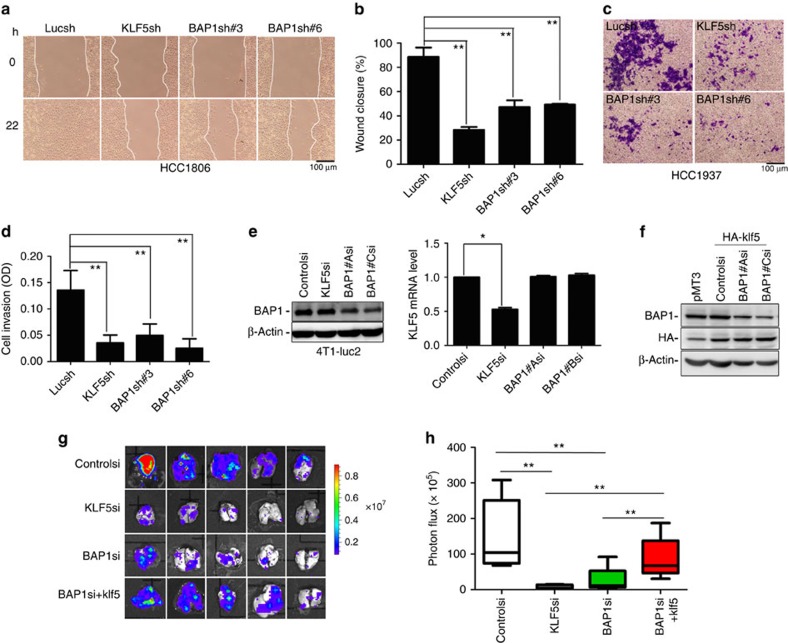
BAP1 knockdown partially suppresses breast cancer lung metastasis through KLF5. (**a**) Knockdown of either BAP1 or KLF5 decreased HCC1806 cell migration as measured by wound-healing assays. The wound edges are indicated by white lines. Representative images are shown. Scale bar, 100 μm. (**b**) The quantitative results of **a** (*n*=3). The *y* axis represents percentage of wound closure. Every experimental group was compared with the Lucsh group. Error bars represent s.d. from three repeats. **P<0.01, *t*-test. (**c**) Knockdown of either BAP1 or KLF5 decreased HCC1806 cell invasion as measured by Matrigel Transwell assays. The blue dye indicated the transwell cells. Representative images are shown. Scale bar, 100 μm. (**d**) The quantitative results of **c**. **P<0.01, *t*-test. The OD values of transwell cells were measured using a microplate reader. Every experimental group was compared with the Lucsh group. Error bars represent s.d. from three repeats. **P<0.01, *t*-test. (**e**) Knockdown of BAP1 in 4T1 cells was confirmed by WB. Knockdown of KLF5 in 4T1 cells was confirmed by RT–quantitative PCR (*n*=3, mean±s.d. from three experiments with triplicate). The KLF5 siRNA group was compared with the control siRNA group. *P<0.05, *t*-test. (**f**) Overexpression of HA-mKlf5 in 4T1 cells was detected by WB. (**g**) Bioluminescence images of mouse lung metastasis from the four groups 5 weeks after orthotopic injection. (**h**) The quantitative results of **g** (*n*=5, each group). The KLF5si or BAP1si groups were compared with the Controlsi group. The rescue group (BAPsi+HA-klf5) was compared with the KLF5si or BAP1si groups. **P<0.01, mean±s.d., *t*-test.

Metastasis is a feature of malignant tumours and a culprit of most cancer-related deaths. Most breast cancer deaths are caused by metastasis to distant organs, including the bone, lung, liver and brain^47^. Somatic mutation of BAP1 has been shown to associate with metastasis in uveal melanoma^39^,^48^. To explore the roles of BAP1 and KLF5 in breast cancer metastasis, we examined breast cancer cell migration and invasion. Knockdown of BAP1 and KLF5 significantly decreased cell migration compared with the Lucsh negative control in HCC1806 and HCC1937 cells using wound-healing and transwell assays (Fig. 6a,b; Supplementary Fig. 6A–C). Consistently, silence of BAP1 or KLF5 expression significantly decreased the invasion of HCC1937 cells in transwell matrigel invasion assays (Fig. 6c,d). Finally, we wondered whether BAP1 and KLF5 promoted metastasis *in vivo*. Pilot experimental results demonstrated that neither HCC1806 nor HCC1937 metastasized to distant vital organs after implanting cancer cells into the mammary fat pads of nude mice. The well-established metastatic breast cancer cell line MDA-MB-231 does not express KLF5 (refs 49, 50) (Supplementary Fig. 4C). Therefore, we chose to use metastatic mouse 4T1-luc2 cells^51^ for the metastatic experiments. BAP1 and KLF5 were transiently knocked down by siRNA in the 4T1 cells (Fig. 6e). The migration of 4T1 cells was significantly inhibited when BAP1 or KLF5 were silenced (Supplementary Fig. 6D–G). Consequently, we injected 4T1 cells orthotopically into the mammary fat pads of BALB/c mice (2 × 10^5^ cells per point, *n*=8). We killed all of the mice on day 32 and found that knockdown of BAP1 or KLF5 significantly decreased lung metastasis according to *ex vivo* bioluminescence imaging (Fig. 5g,h). Transient KLF5 overexpression in 4T1 cells partially rescued BAP1 knockdown-induced metastasis inhibition (Fig. 6f–h; Supplementary Fig. 7A–C). We repeated the metastasis experiment in 4T1 cells using stable overexpression of human KLF5-3 × Flag and obtained similar results (Supplementary Fig. 7D–F). Therefore, these findings clearly indicate that BAP1 promotes breast cancer lung metastasis partially through stabilizing KLF5.

## Discussion

The transcription factor KLF5 has been well documented to promote cancer cell cycle progression, survival, migration, stemness and tumorigenesis^22^,^52^,^53^,^54^. Given the oncogenic role of KLF5 in a number of cancers including breast, colon and bladder^22^,^23^,^55^, a more thorough understanding of the KLF5 upstream regulatory mechanism may provide novel therapeutic targets for KLF5-positive cancers. Our previous studies have demonstrated that KLF5 is an unstable protein with a short half-life^26^ and that two E3 ubiquitin ligases, WWP1 and SCF^Fbw7^, target KLF5 for ubiquitin-proteasomal degradation^28^,^29^. YAP and TAZ antagonize WWP1-mediated KLF5 degradation^56^,^57^. It is well known that ubiquitination can be reversed by DUBs. Loss-of-function screening with genome-scale RNA interference libraries has been demonstrated to be a powerful tool for identifying novel regulators^58^,^59^. We performed a genome-wide loss-of-function screening and identified BAP1 as a DUB for KLF5.

Several lines of evidence support that BAP1 is the KLF5 DUB. First, BAP1 interacts with KLF5 directly. Second, BAP1 decreases KLF5 polyubiquitination and increases KLF5 protein stability in a DUB activity-dependent manner. In addition, the functions of BAP1 and KLF5 are similar in terms of promoting cell cycle progression, migration, tumour growth and lung metastasis. Finally, BAP1 promotes breast tumour growth and metastasis partially through KLF5, because KLF5 overexpression partially rescued the BAP1 knockdown-induced tumour growth and metastasis inhibition.

Accumulated genetic evidence suggests that BAP1 could be a tumour suppressor because the *BAP1* gene is ubiquitously mutated in mesothelioma, uveal melanoma, melanocytic tumour, renal cell carcinoma and other cancers^31^,^34^. However, BAP1 is rarely mutated in breast cancers^36^,^60^. We confirmed that BAP1 is not mutated in HCC1806 and HCC1937 by complementary DNA sequencing. BAP1 may play a critical role in maintaining genome stability and DNA damage repair^61^,^62^,^63^. Ventii *et al*.^31^ reported that BAP1 overexpression in the BAP1-null NCI-H226 human non-small-cell lung cancer cell line suppressed growth in DUB activity- and nuclear localization-dependent manners by accelerating the cell cycle G1/S transition and inducing cell death. BAP1 overexpression also suppressed cell growth in the 769-P renal cancer cell line *in vitro*^41^. In sharp contrast, Machida. *et al*.^33^ demonstrated that BAP1 knockdown in MCF10A suppressed cell growth in a DUB activity-dependent manner, while BAP1-C91S overexpression inhibited cell growth by interacting with HCF-1. Similarly, Bott *et al*.^37^ showed that BAP1 overexpression in BAP1-null malignant pleural mesothelioma cell lines promoted cell proliferation and that BAP1 knockdown decreased proliferation in three malignant pleural mesothelioma cell lines. In this study, we showed that BAP1 knockdown decreased DNA synthesis, the number of S-phase cells and cell growth in HCC1806 cells. Importantly, BAP1 stable knockdown suppressed tumour growth in HCC1806 cells and inhibited lung metastasis in 4T1 cells. In both models, BAP1 knockdown-induced growth and metastasis inhibition was partially rescued by overexpression of KLF5 (Figs 5 and 6; Supplementary Fig. 7). We did not detect a full rescue because overexpression of KLF5 in HCC1806 and 4T1 cells is not completely efficient (Figs 5 and 6). How BAP1 and KLF5 promote metastasis requires further investigation. Nevertheless, our results suggest that BAP1 promotes breast tumour growth and metastasis partially through stabilizing KLF5. It is also possible that BAP1 has other substrates, such as HCF-1 and BRCA1. BAP1 and KLF5 promote breast cancer migration, invasion and metastasis may not through p27; however, KLF5 is a transcription factor regulating numerous downstream target genes, such as FGF-BP and mPGES1. Taken together, BAP1 may have a context-dependent function in terms of regulating tumour initiation and progression. The physiological and pathological functions of BAP1 require further investigation using transgenic mouse models. Dey *et al*.^42^ generated BAP1 knockout mice using a tamoxifen-inducible system, and found that the mice recapitulate features of human myelodysplastic syndrome.

KLF5 may regulate the transcription of a subset of target genes together with the BAP1/HCF-1 protein complex. When KLF5 or BAP1 were immunoprecipitated, HCF-1 and OGT proteins were co-immunoprecipitated (Fig. 4). Indeed, depletion of KLF5, BAP1, HCF-1 or OGT upregulated p27 protein expression and blocked cell cycle progression (Fig. 4). Depletion of KLF5, BAP1 or HCF-1 also upregulated *p27* mRNA levels (Fig. 4). Interestingly, depletion of OGT increased p27 protein levels, but not mRNA levels. It has been reported that OGT depletion decreases the expression levels of FoxM1 and its target gene Skp2 (ref. 64), which is an E3 ligase for p27 (ref. 65). It appears that OGT promotes p27 protein degradation but does not increase its gene transcription in HCC1806 cells. Finally, both KLF5 and BAP1 bind to the *p27* gene promoter. These findings suggest that KLF5, BAP1 and HCF-1 may form a transcriptional complex to regulate target gene transcription.

Since high KLF5 mRNA and protein levels have been reported as a potent biomarker for unfavourable prognosis for breast cancer patients, we tried to test whether BAP1 can also serve as a biomarker for prognosis for breast cancer patients. Unfortunately, two commercially available anti-BAP1 antibodies did not work for immunohistochemical staining in breast tumours. We analysed the *BAP1* mRNA expression levels in breast tumours based on the TCGA database. The expression level of *BAP1* mRNA in breast tumours is not associated with patient overall survival (Supplementary Fig. 8A) and distant metastasis-free survival (Supplementary Fig. 8B). Interestingly, a high level of *BAP1* mRNA is associated with long relapse-free survival (Supplementary Fig. 8C). This is opposite to KLF5 (Supplementary Fig. 8D). However, BAP1 positively regulates KLF5 at the protein level. Therefore, it is important to develop a good antibody examining the BAP1 protein expression in breast tumours by immunohistochemical staining in the future.

In conclusion, BAP1 is a KLF5 DUB and BAP1 promotes basal breast cell proliferation, migration, tumour growth and metastasis partially through stabilizing KLF5 (Supplementary Fig. 9). In this study, we discovered a novel positive regulatory mechanism for KLF5 and the functional mechanism by which BAP1 promotes breast cancer growth and metastasis. Our findings imply that BAP1 could serve as a potential therapeutic target in basal-like breast cancer.

## Methods

### DUB siRNA library screening

The siRNA library consisting of 87 human DUBs was purchased from Applied Biosystems (Ambion Silencer siRNA Custom Library, catalogue number 4392425).

Different siRNAs were transfected with LF2000 (Invitrogen, #11668-019) into HeLa cells in 24-well plates for 2 days. Cell lysates were extracted and the protein levels of endogenous KLF5 were measured by WB. We identified 27 candidates in the first round of screening. The candidate genes were further validated in MCF10A and HeLa cells.

### Antibodies

All primary antibodies were diluted 1,000 times for WB if not specified. The KLF5 rabbit polyclonal antibody has been described in a previous study^29^. The anti-BAP1 mouse monoclonal (sc-28383), rabbit polyclonal (sc-28236), anti-HA (Y11) rabbit polyclonal (sc-805), anti-His (H-15) rabbit polyclonal (sc-803), anti-Myc (9E10) mouse monoclonal (sc-40) and anti-GAPDH (FL-335) rabbit polyclonal (sc-25778) antibodies were purchased from Santa Cruz Biotechnology (Santa Cruz, CA). The anti-OGT (DM-17) rabbit polyclonal (#O6264), anti-Flag rabbit polyclonal (F7425), anti-GST rabbit polyclonal (G7781) and anti-β-actin mouse monoclonal (A5441, 1:10,000 dilution) antibodies were from Sigma-Aldrich (St Louis, MO). The goat anti-KLF5 and mouse anti-FGF-BP monoclonal antibodies were from R&D Systems (Minneapolis, MN). The mouse anti-p27 (#610241) monoclonal antibody was from BD Biosciences. The rabbit anti-HCF-1 (A301-399A) polyclonal antibody was from Bethyl Laboratories. The anti-WWP1 (H00011059-M01) rabbit polyclonal antibody was from Abnova and the mouse anti-A20 (#60-6629) monoclonal antibody was from eBioscience. The mouse anti-ubiquitin (#MAB1510) monoclonal antibody was from Millipore. The anti-K48-polyubiquitin (#8081) and anti-K63-polyubiquitin (#5621) rabbit polyclonal were purchased from Cell Signaling. Some important original immunoblotting results are shown in supplementary Fig. 11.

### Cell culture and transfection

All cell lines were purchased from American Type Culture Collection (Manassas, VA, USA). The HeLa cervical cancer and HEK293FT human embryonic kidney and MDA-MB-468 human breast cancer cell lines were cultured in DMEM (high glucose) medium containing 10% fetal bovine serum (FBS, Hyclone) and 1% penicillin/streptomycin (P/S). The human breast cancer cell lines HCC1806 and HCC1937 and the mouse breast cancer cell line 4T1 were maintained in RPMI-1640 containing 10% FBS and 1% P/S. The SUM149PT breast cancer cell line was grown in HAM/F1 media supplemented with 10% FBS, 0.005 mg ml^−1^ insulin, 1 μg ml^−1^ hydrocortisone, 10 mM HEPES and 1% P/S. The MDA-MB-231 breast cancer cell was cultured in DMEM/F12 medium with 10% FBS and 1% P/S. The immortalized breast epithelial cell line MCF10A was maintained in DMEM/Ham's F12 50/50 medium supplemented with 5% horse serum, 0.5 μg ml^−1^ hydrocortisone, 10 μg ml^−1^ insulin, 20 ng ml^−1^ epidermal growth factor, 0.1 μg ml^−1^ cholera enterotoxin, 100 U ml^−1^ penicillin and 100 μg ml^−1^ streptomycin (P/S), and 2 mM L-glutamine^66^. All of the siRNAs and plasmids were transfected using either LF2000 or the X-tremeGENE HP DNA Transfection Reagent (Roche, #06366236001). All siRNA sequences are shown in Supplementary Table 1.

### Recombinant protein expression and purification

Human BAP1 (WT) plasmid was subcloned from MCF10A cells (primers are shown in Supplementary Table 2). The BAP1-C91S was generated using the PCR-directed mutagenesis method. BAP1 and BAP1-C91S were subcloned into the prokaryotic GST-fused expression vector pGEX-6p-1. The constructs were transformed into the *E. coli* strain BL21 (DE3) and 1 mM isopropyl-b-D-thiogalactoside was added to induce recombinant protein expression after the overnight incubation at 15 °C. The cells were harvested, resuspended in 50 ml lysis buffer containing 100 mM Tris-HCl, pH 8.0, 100 mM NaCl, 50 mM EDTA, 2% Triton X-100, 1.2 μg ml^−1^ phenylmethylsulphonyl fluoride, 5 mM dithiothreitol and 1.67 mg ml^−1^ lysozyme for 30 min and sonicated. The GST fusion proteins were affinity purified with glutathione sepharose 4B and eluted with elution buffer (50 mM Tris-HCl, pH 8.0 and 10 mM glutathione). The proteins were analysed by 10% SDS–polyacrylamide gel electrophoresis with Coomassie blue staining.

KLF5 was subcloned into the pET28-3C vector. The plasmid was transfected into BL21 (DE3) cells to make protein as described above. Next, the cells were harvested in lysis buffer (20 mM Tris, pH 8.0, 0.2 mM β-mercaptoethanol and 500 mM NaCl) and purified with a Ni-NTA (Qiagen) column.

### K48-linked and K63-linked polyubiquitin chains

The polyubiquitin chains (Ub_3–7_, K48-linked) (Cat.#UC-220) and the polyubiquitin chains (Ub_3–7_, K63-linked) (catalogue number UC-320) were purchased from Boston Biochem. The peptides were dissolved in aqueous buffer and the final concentration is 2 mg ml^−1^.

### Protein–protein interaction assays

The GST pull-down assay has been described in a previous study^28^. For the nickel beads pull-down assays, purified KLF5-6 × His and GST-BAP1 were incubated with Ni-NTA beads at 4 °C, rocking gently overnight. Following incubation, the beads were washed three times with 1 ml lysis buffer (20 mM Tris, pH 8.0, 0.2 mM β-mercaptoethanol and 500 mM NaCl). Finally, the samples were resuspended in SDS sample buffer and analysed by WB.

### Deubiquitination assays

The ubiquitination assay was performed in our previous study^67^. The *in vitro* deubiquitination assay is described here. HEK293FT cells were transfected with HA-Ub, KLF5-3 × Flag and Myc-Fbw7γ. At 2 days after transfection, the cells were treated with 20 mM MG132 for 6 h to enrich the polyubiquitinated KLF5 proteins. Then, Flag-tagged polyubiquitinated KLF5 proteins were purified by immunoprecipitation with Flag-M2 beads and eluted using the 3 × Flag peptide (100 μg ml^−1^, Sigma). Next, the purified polyubiquitinated KLF5 proteins were incubated with GST, GST-BAP1 or GST-BAP1-C91S in deubiquitination buffer (50 mM Tris-HCl, pH 8.0, 100 mM NaCl, 5 mM MgCl_2_, 1 mM adenosine triphosphate and 1 mM dithiothreitol) for 6 h at 37 °C. The reaction was terminated by adding SDS sample buffer. Ubiquitinated KLF5 was detected by WB using the anti-HA antibody.

### Animals

Twenty-four 3–4-week-old female NOD-SCID mice were purchased from Vital River (Beijing, China). Twenty-four 5–6-week-old female BALB/c nude mice were purchased from Slaccas (Changsha, Hunan, China). The animal protocol was approved by the animal ethics committee of Kunming Institute of Zoology, CAS.

### Cell proliferation *in vitro* and tumour growth *in vivo*

HCC1806 cell proliferation was measured using Click-iT EdU Alexa Fluor 488 imaging kits (Invitrogen) and the sulforhodamine B assay was used for measuring cell viability as described previously^66^. Twenty-four NOD-SCID mice were haphazardly distributed into four groups (Lucsh, BAP1#3sh, BAP1#6sh and KLF5sh; six mice per group). Twenty-four nude mice were used for the second experiment. Five million HCC1806 cells were resuspended in Matrigel (BD Biosciences; 1:1 diluted with PBS) and subcutaneously injected into the fourth pair of mammary gland fad pads. Tumour sizes were measured twice a week and calculated using the equation: 1/2 × length × width^2^. All of the mice were killed at the end and tumours were harvested and weighed.

### Cell migration and invasion assays

HCC1806, HCC1937 and 4T1 cells were plated in 12-well plates for wound-healing assays. For the Transwell assays (Costar, #3422), the cells were placed on the upper layer of a cell-permeable membrane and media containing 10% FBS was placed in the lower chamber. Following an incubation period, the cells that had migrated through the membrane were stained and counted. The membrane was also coated with matrigel (1:4 dilution ratio) for testing invasion.

### Tumour metastasis in BALB/c mice

Briefly, the nude mice were anesthetized with ketamine and then 2 × 10^5^ 4T1 cells in 50 μl PBS with 50 μl Matrigel (10 mg ml^−1^) were injected into the mammary fat pads. The mice were imaged to test for metastasis.

### Statistical analysis

All experiments were repeated three times and were analysed as the mean±s.d. When every experimental group is compared with the control group, one-way analysis of variance was used to compare means of groups; then Dunnet's *t*-test was used to compare each group with the control group. The SPSS software V.19.0 is used for all the analysis. *P* values less than 0.05 were considered to be statistically significant.

## Additional information

**How to cite this article:** Qin, J. *et al*. BAP1 promotes breast cancer cell proliferation and metastasis by deubiquitinating KLF5. *Nat. Commun*. 6:8471 doi: 10.1038/ncomms9471 (2015).

## Supplementary Material

Supplementary Information Supplementary Figures 1-11 and Supplementary Tables 1-2
