# Supplementary material for: BAP1 promotes breast cancer cell proliferation and metastasis by deubiquitinating KLF5
Source: Nat Commun. 2015 Sep 30;6:8471. doi: 10.1038/ncomms9471 (PMC4598844; doi:10.1038/ncomms9471)
Supplement: Supplementary Information — Supplementary Figures 1-11 and Supplementary Tables 1-2 [file ncomms9471-s1.pdf]

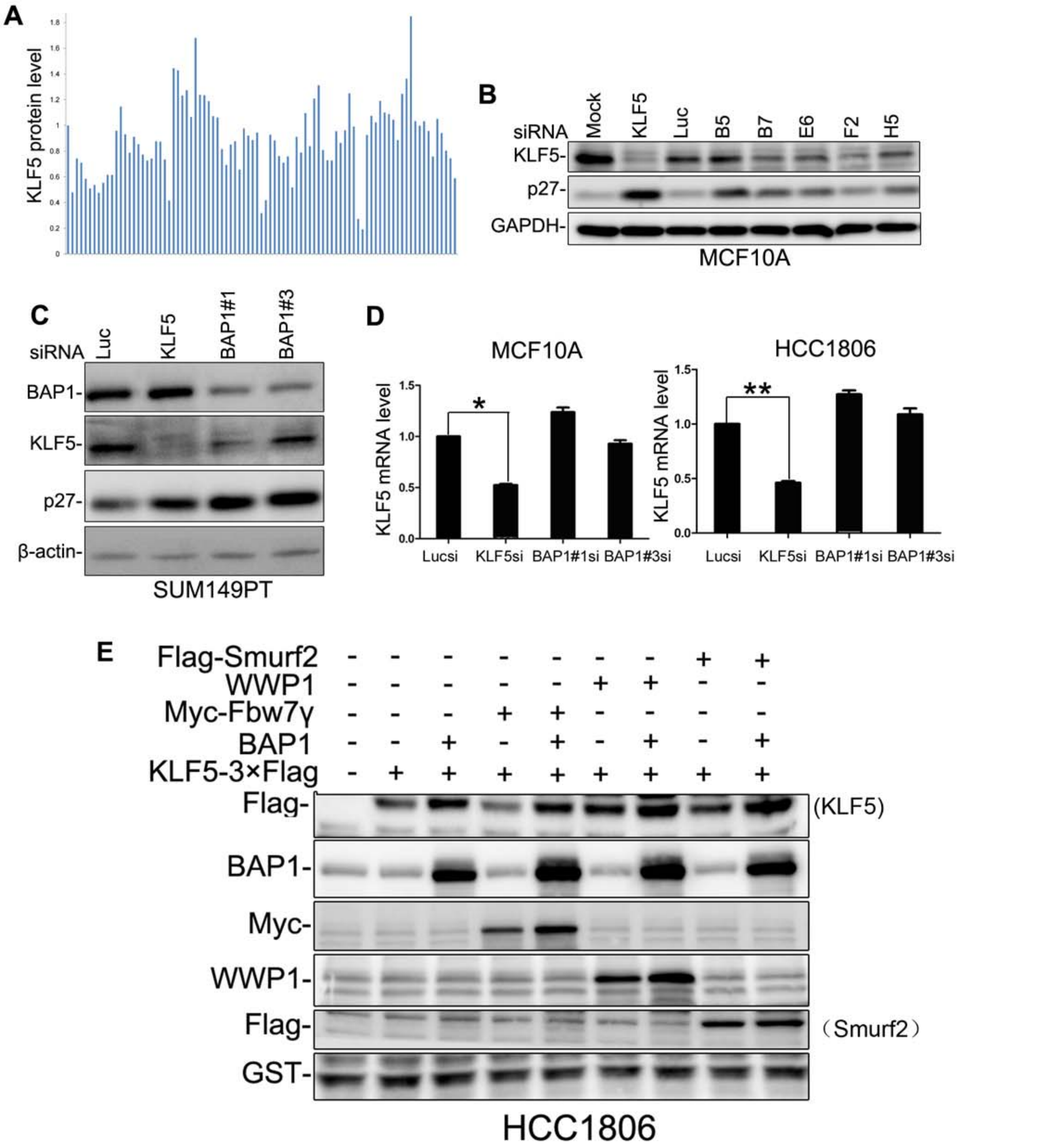

Supplementary Figure 1. Validation of BAP1 as a KLF5 DUB

A. KLF5 protein levels were measured after DUBs were silenced by the siRNA library in HeLa cells. The DUB siRNA was individually transfected into HeLa cells for 48 h and the KLF5 protein level was detected by WB. The KLF5 band intensities were quantification by the Image J software.

B. Validation of 5 candidate KLF5 DUBs in MCF10A cells. The DUB siRNA was individually transfected into MCF10A cells for 48 h and the KLF5 and p27 protein levels were detected by WB.

C. Knockdown of endogenous BAP1 in SUM149PT cells decreased the KLF5 protein levels and increased the p27 protein levels.

D. BAP1 knockdown did not decrease the KLF5 mRNA levels in MCF10A and HCC1806 cells as detected by RT-qPCR. The KLF5 mRNA levels were normalized to β-actin (mean±SD from three experiments in triplicate). Every experimental group (KLF5si, BAP1#1si, BAP1#3si) was compared with the Lucsi group, *$p < 0.05$, **$p < 0.01$.

E. BAP1 antagonized E3 ligase-mediated degradation of KLF5. HCC1806 cells were transfected withthe BAP1 or KLF5 E3 ligases (WWP1, Fbw7γ and Smurf2) for 48 h. The cell lysates were analyzed by WB.

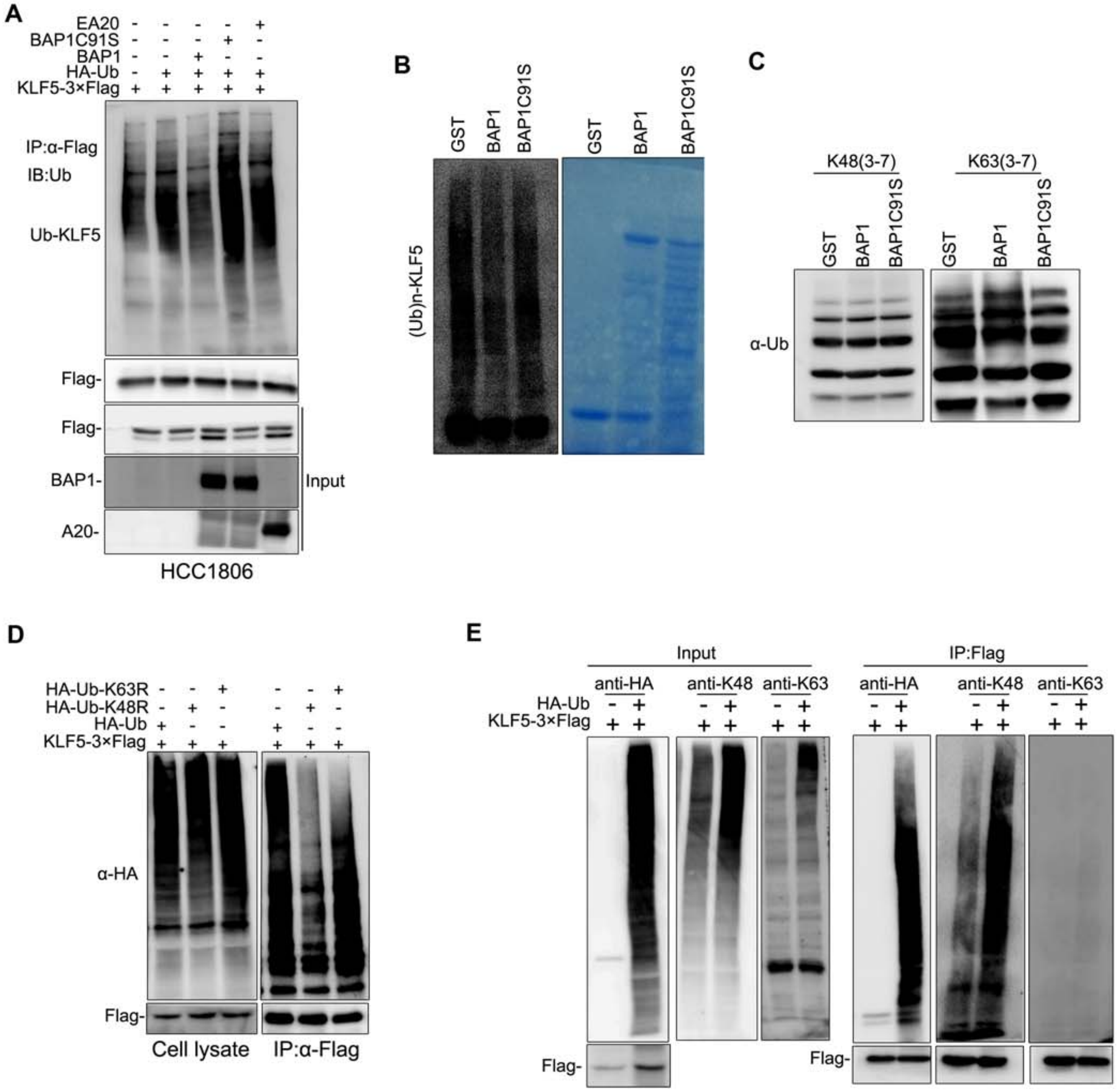

Supplementary Figure 2. BAP1 is a KLF5 DUB removing the K48-linked polyubiquitin chains from KLF5.

A. BAP1 decreased KLF5 polyubiquitination in HCC1806 cells. KLF5-3 Flag and HA-Ub were co-expressed with BAP1, BAP1-C91S or A20. After the cells were treated with MG132 for 6 h, KLF5 proteins were immunoprecipitated and the polyubiquitinated KLF5 proteins were detected by WB using an anti-Ub antibody.

B. BAP1 deubiquitinates KLF5 in vitro. Ubiquitinated KLF5 was purified from MG132-treated HEK293FT cellsand then incubated with purified GST-tagged BAP1 or BAP1-C91S in vitro. The polyubiquitinated KLF5 proteins were examined by WB using the anti-HA antibody.

C. BAP1 did not degrade free K48-linked polyubiquitin chains (K48-linked (3-7) poly-Ub) or K63-linked polyubiquitin chains (K63-linked (3-7) poly-Ub). Polyubiquitin chains were incubated with purified GST, GST-BAP1, or GST-BAP1-C91S under the deubiquitination assay.

D. KLF5 polyubiquitination is predominately K48-linked. HEK293FT cells were transfected with KLF5-3×Flag and HA-Ub or its mutants (K48R and K63R) to assess the linkage of KLF5 polyubiquitin chains.

E. HA-Ub and KLF5-3×Flag were transfected into HEK293FT cells. The ubiquitination of KLF5 was detected by linkage-specific antibodies that recognize K48- or K63-linked polyubiquitin chains.

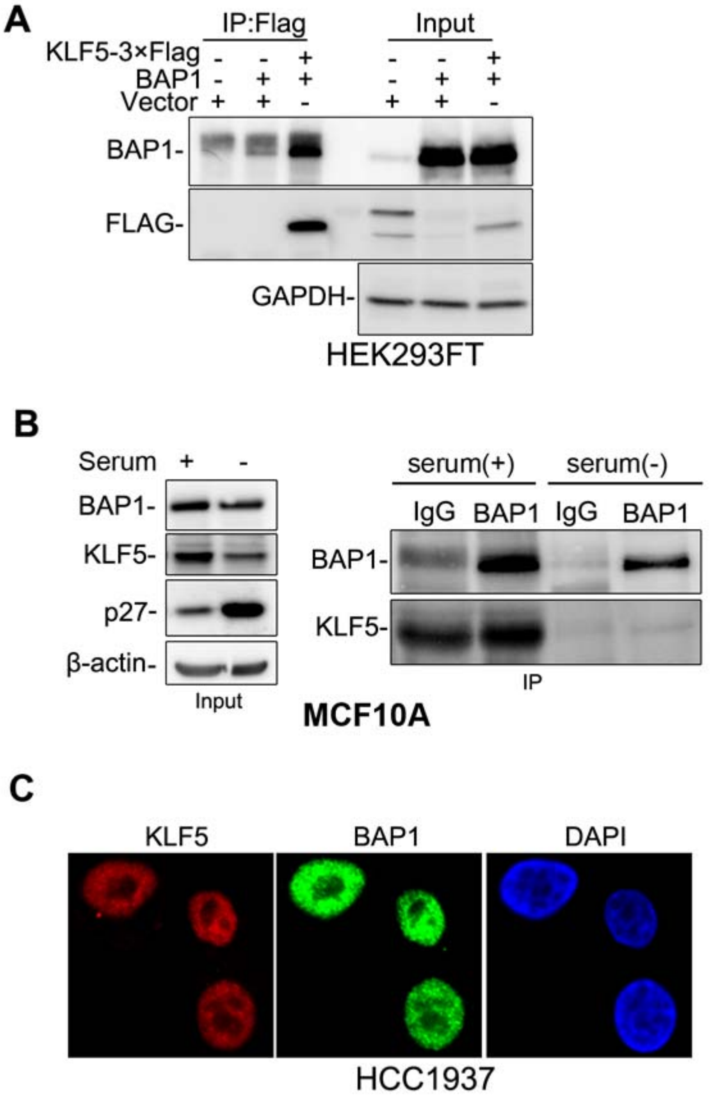

Supplementary Figure 3. BAP1 and KLF5 proteins interact with each other

A. Exogenous BAP1 and KLF5 proteins interact with each other. KLF5-3×Flag and BAP1 were co-transfected into HEK293FT cells. KLF5-3×Flag was immunoprecipitated with the Flag M2 beads and BAP1 was specifically co-immunoprecipitated.

B. Serum increased the interaction between BAP1 and KLF5. MCF10A cells were serum starved for 24 h or cultured in the media with 10% serum. Cell lysate was collected for immunoprecipitation using the anti-BAP1 antibody. Serum upregulated the KLF5 and BAP1 protein levels and downregulated the p27 protein level.

C. BAP1 and KLF5 are co-localized in the nucleus of HCC1937 cells. The cellular location of KLF5 and BAP1 was examined by immunofluorescence staining. DAPI was used to stain nuclei.

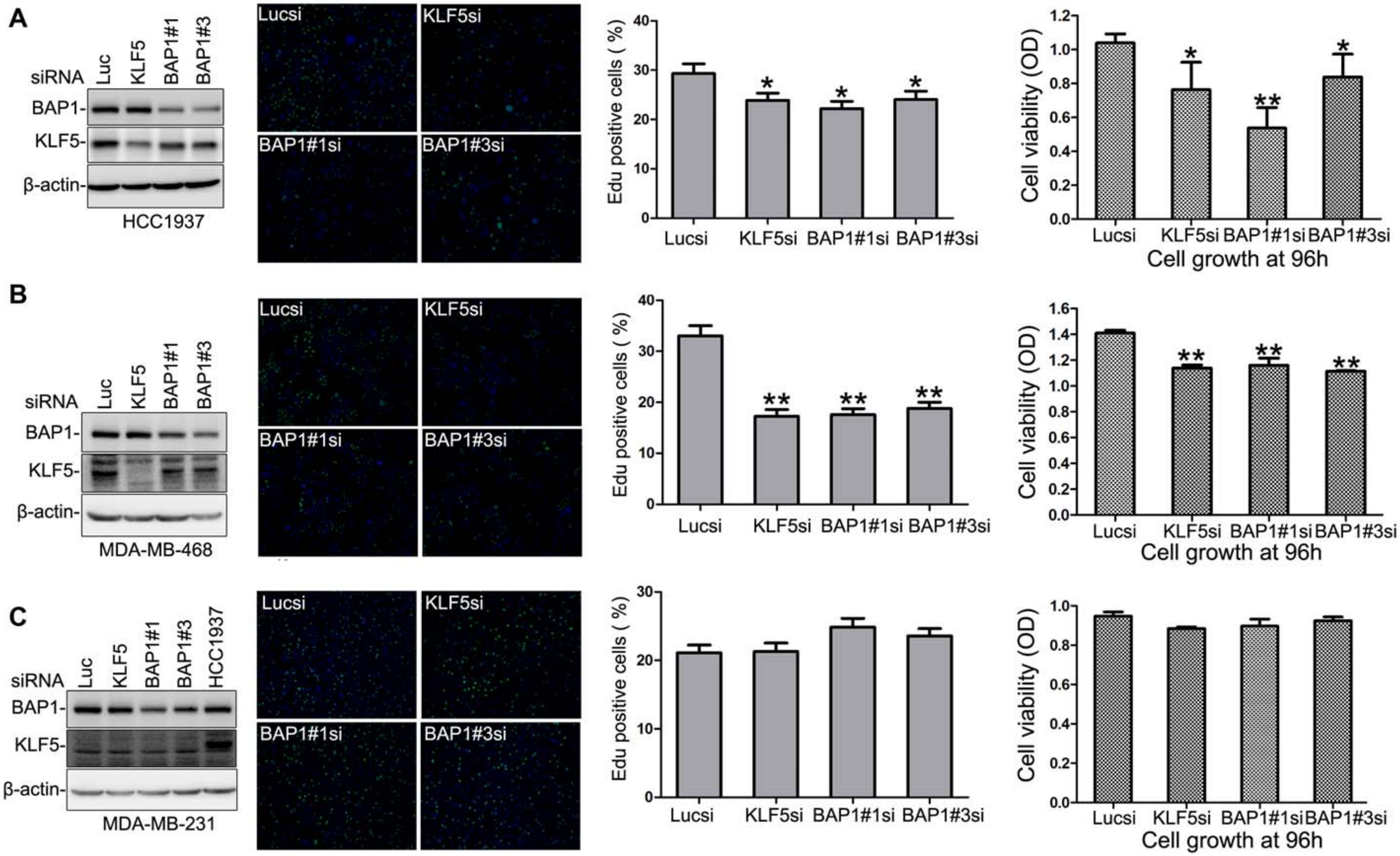

Supplementary Figure 4. Knockdown of BAP1 inhibits DNA synthesis and cell growth in KLF5 positive breast cancer cell lines

A. Knockdown of BAP1 and KLF5 inhibited DNA synthesis and cell growth in HCC1937. BAP1 was knocked down using two different siRNAs. The DNA synthesis and cell growth were significantly decreased when KLF5 or BAP1 were knocked down. Every experimental group (KLF5si, BAP1#1si and BAP1#3si) was compared with the Lucsi group, *p <0.05, **p <0.01.

B. Knockdown of BAP1 and KLF5 inhibited DNA synthesis and cell growth in MDA-MB-468.

C. Knockdown of BAP1 and KLF5 did not inhibit DNA synthesis and cell growth in MDA-MB-231, which is a KLF5 negative breast cancer cell line.

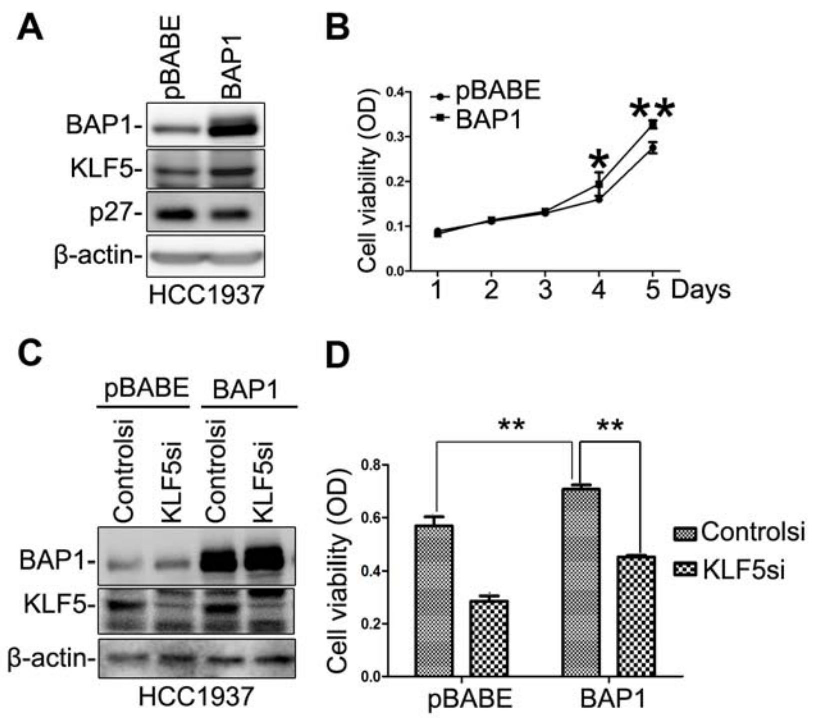

Supplementary Figure 5. Overexpression of BAP1 in HCC1937 cells promotes cell growth through KLF5 in part

A. Stable overexpression BAP1 in HCC1937 cells increased KLF5 and decreased p27 protein levels.

B. Stable overexpression of BAP1 promoted cell growth in vitro, as measured by the SRB assay. HCC1937 cells stably overexpressing pBABE or BAP1 were planted in 24 wells. Cells growth was measured every day. Data points represent the mean ± SD of three duplicates per group. Statistical significance was determined by t-test. The BAP1 group was compared with the pBABE group. *p <0.05, **p <0.01.

C. KLF5 was transiently knockdown by siRNAs in BAP1 stable overexpression HCC1937 cells and control cells. The BAP1 and KLF5 protein levels were detected by WB.

D. Depletion of KLF5 blocked BAP1 overexpression-induced cell growth.

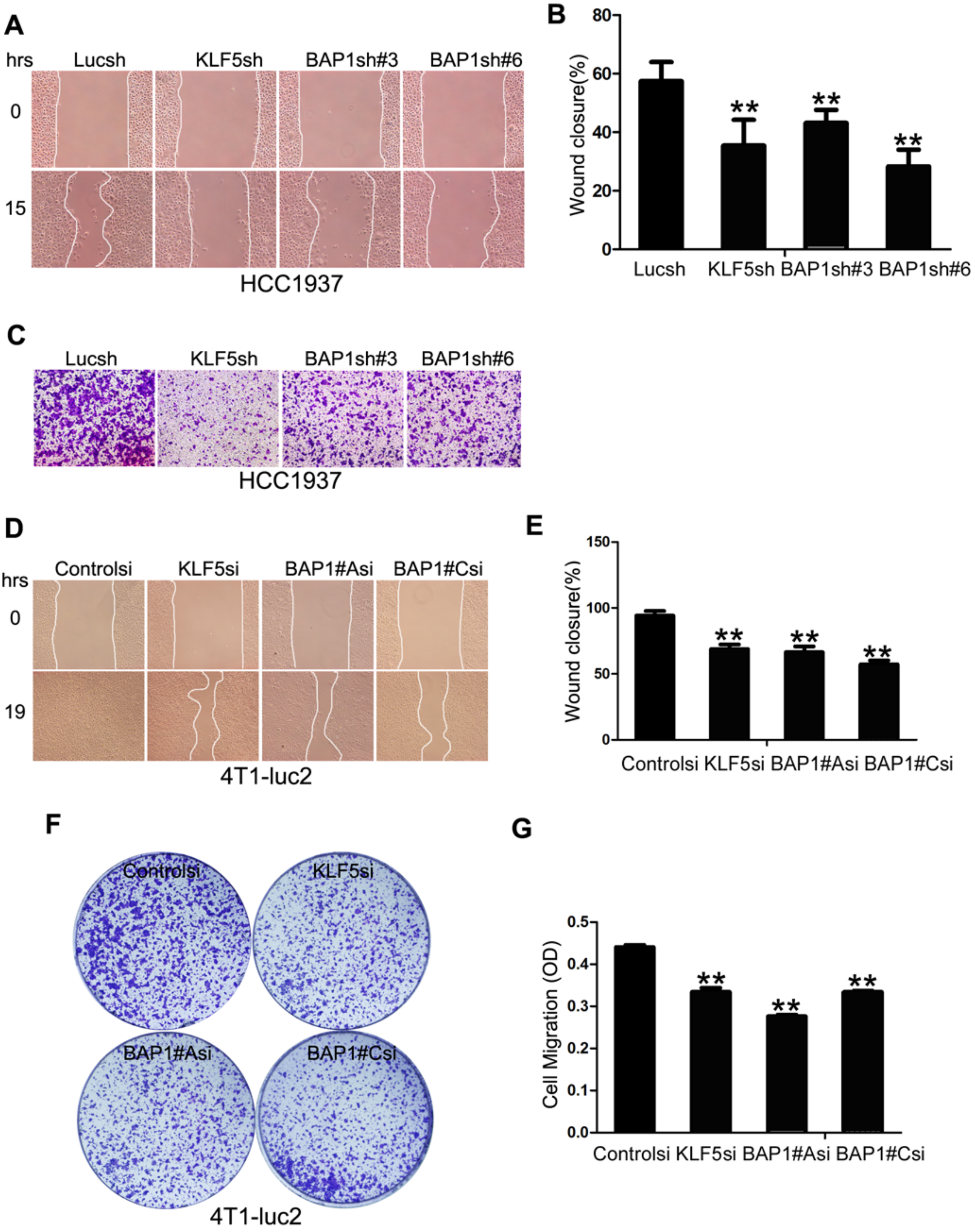

Supplementary Figure 6. BAP1 and KLF5 knockdown inhibits cell migration in HCC1937 and 4T1 cells

A. Knockdown of either BAP1 or KLF5 decreased HCC1937 cell migration as measured by wound-healing assays.

B. The quantitative results of panel A. The y-axis represents percentage of wound closure. Every experimental group was compared with the Lucsh group, **p <0.01.

C. Stable knockdown of either BAP1 or KLF5 decreased HCC1937 cell migration as measured by transwell assays.

D. Transient knockdown of either BAP1 or KLF5 decreased 4T1 cell migration as measured by wound-healing assays.

E. The quantitative results of panel D. The y-axis represents percentage of wound closure. Every experimental group was compared with the Controlsi group, **p <0.01.

F. Transient knockdown of either BAP1 or KLF5 decreased 4T1 cell migration as measured by transwell assays.

G. The quantitative results of panel F. The OD values of transwell cells were measured using a microplate reader. Every experimental group was compared with the Controlsi group (n=3). **p <0.01.

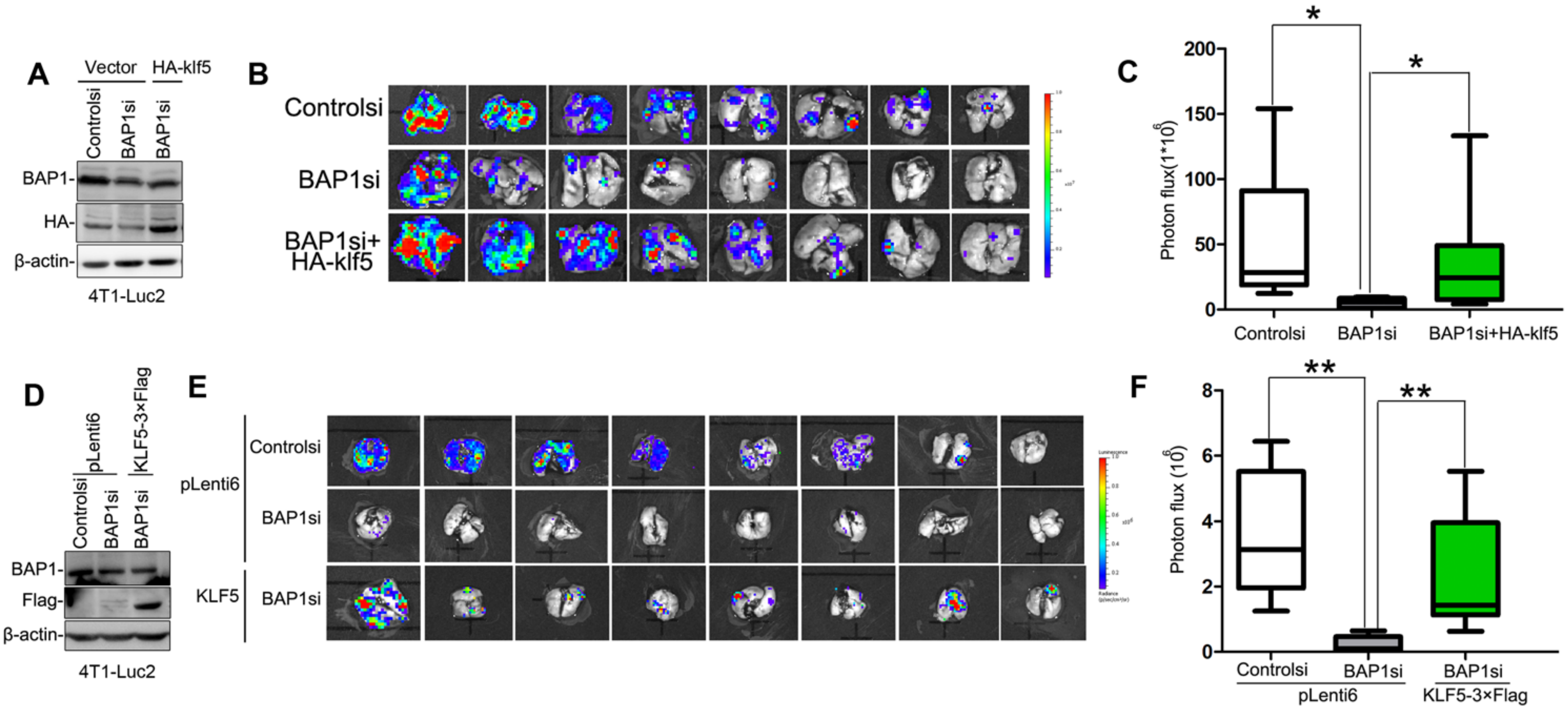

Supplementary Figure 7. Overexpression of KLF5 partially rescued the 4T1 cell lung metastasis decrease induced by BAP1 depletion

A. Transient overexpression of HA-mKlf5 in 4T1 cells was detected by WB.

B. Bioluminescence images of mouse lung metastasis from the three groups 5 weeks after orthotopic injection.

C. The quantitative results of panel B (n=8, each group). The BAP1si group was compared with the Controlsi group and the rescue group (BAPsi+HA-klf5). *p <0.05.

D. Stable overexpression KLF5-3×Flag in 4T1-luc2 cells. Then, BAP1 was transiently knocked down in KLF5 overexpression cells and control cells.

E. Bioluminescence images of mouse lung metastasis from three groups 4 weeks after orthotopic injection.

F. The quantitative results of panel E (n=8, each group). The BAP1si group was compared with the Controlsi group and the rescue group (BAPsi+ KLF5-3×Flag). **p <0.01.

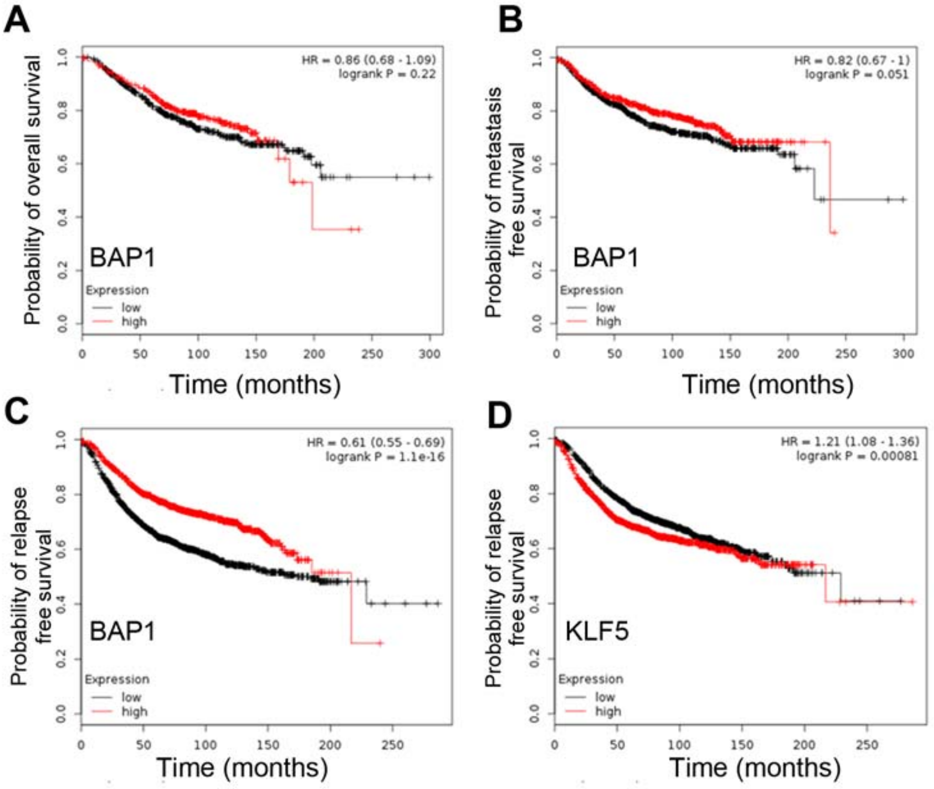

Supplementary Figure 8. The clinical relevance of BAP1 mRNA expression in breast tumors from the TCGA database

A. The expression level of BAP1 mRNA in breast tumors is not associated with patient overall survival. Kaplan-Meier plotter was used to analyze the breast cancer RNA-seq data from the TCGA database.

B. The expression level of BAP1 mRNA in breast tumors is not associated with patient metastasis free survival.

C. High expression level of BAP1 mRNA in breast tumors is significantly ($p<0.01$) associated with long patient relapse free survival.

D. High expression level of KLF5 mRNA in breast tumors is significantly ($p<0.01$) associated with short patient relapse free survival.

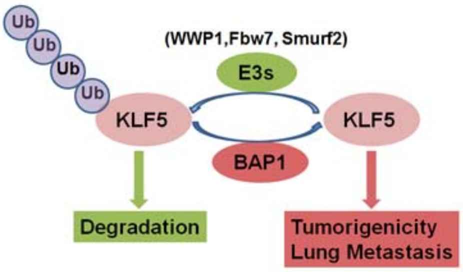

Supplementary Figure 9. Working model about BAP1 and KLF5 functions in tumorigenicity and lung metastasis

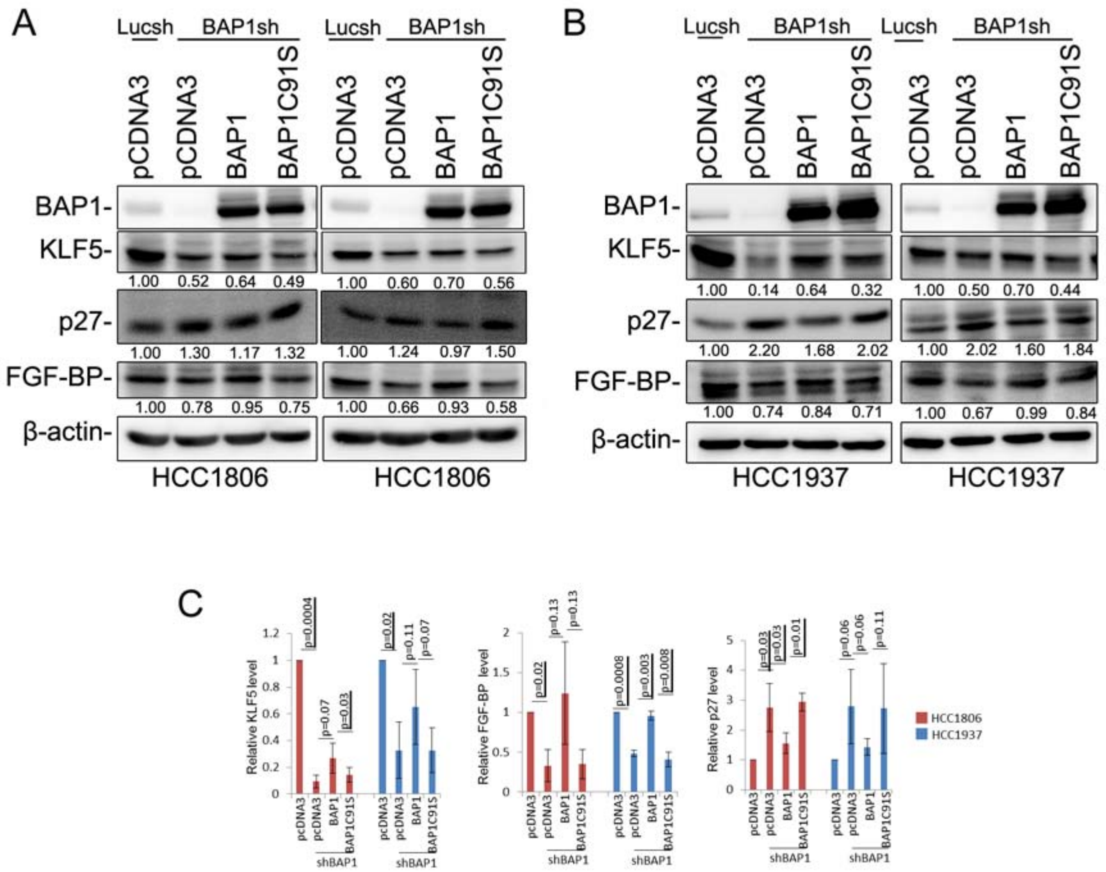

Supplementary figure 10. BAP1 stable knockdown by shRNA#6 induced KLF5, FGF-BP and p27 protein expression changes were rescued by transient overexpression of BAP1, but not BAP1C91S in HCC1806 and HCC1937 cells. We repeated these experiments for extra two times and obtained similar results (A&B). We performed the statistical analysis based on our quantified normalized band intensities for KLF5, FGF-BP and p27. The expression change trends of KLF5, FGF-BP and p27 are consistent with our hypothesis that BAP1 stabilizes KLF5 and promotes its transactivation functions through its enzyme activity. Ten p values (underlined) are less than 0.05, however eight p values are larger than 0.05 because of large variations (SD values are large). The reproducibility and the average results of three completely independent experiments in two different cell lines (HCC1806 and HCC1937) support the same conclusion that BAP1 stabilizes KLF5 dependent on its enzyme activity.

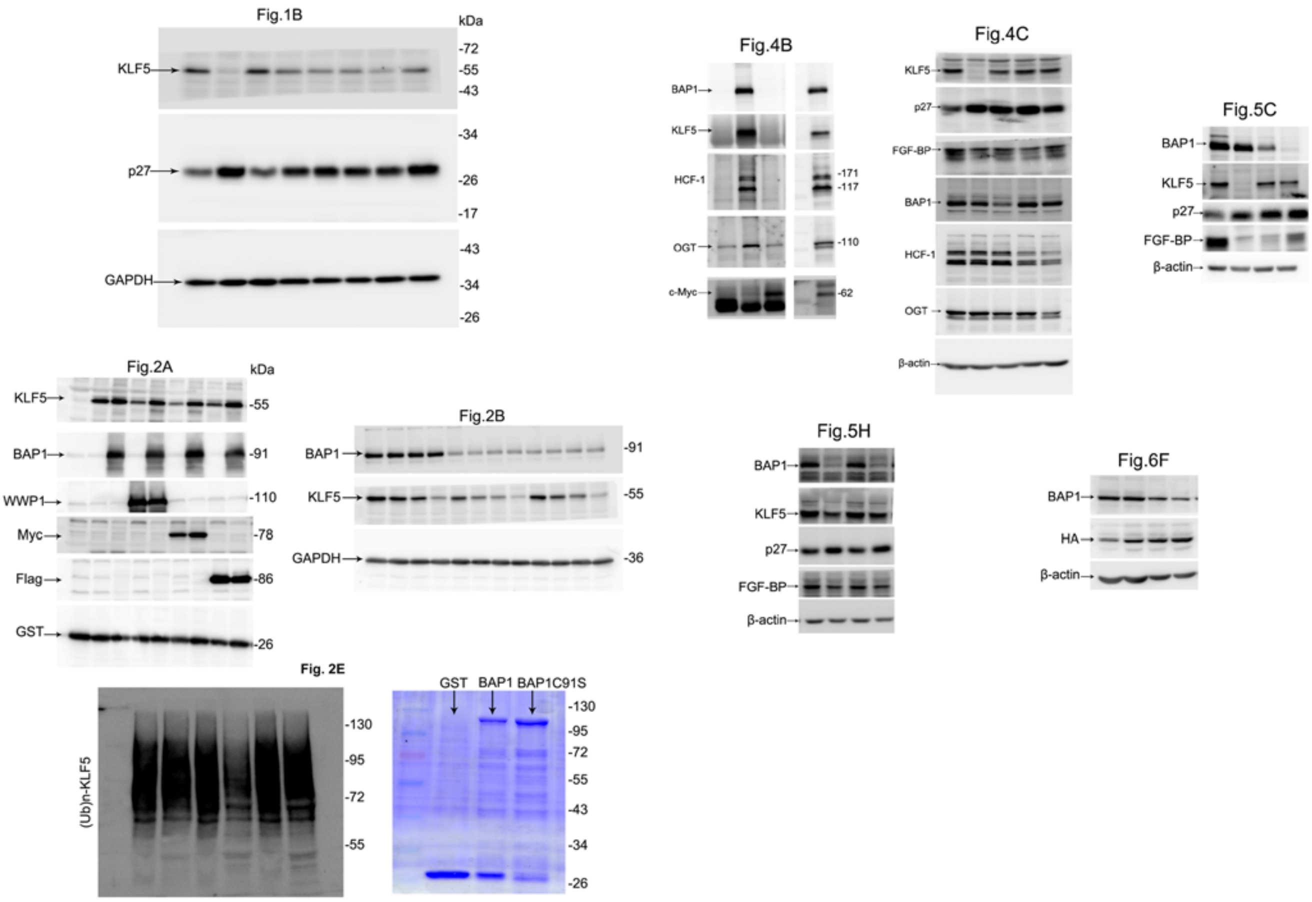

Supplementary figure 11. Original immunoblotting results for figures 1B, 2A-B, 2E, 4B-C, 5C, 5H and 6F.

SUPPLEMENTARY TABLES

Supplementary Table 1: siRNA and shRNA Oligonucleotides Used in This Study

**Target genes**	**Sequences (5' to 3')**
Luciferase siRNA/shRNA	CTTACGCTGAGTACTTCGA
*KLF5*siRNA/shRNA (human)	GAUUACCCUGGUUGCACA
*KLF5*siRNA (mouse)	AAGCUCACCUGAGGACUCA
BAP1#1siRNA (human)	CGUCCGUGAUUGAUGAUGA
BAP1#3 siRNA /shRNA (human)	CCAACUCUUGUGCAACUCA
*BAP1#6*shRNA (human)	AGAGCAAAGGATATGCGAT
*BAP1#A*siRNA (mouse)	GCUUCAAGAGUCUCAGAAA
*BAP1#C*siRNA (mouse)	CCAACCUAGUGGAACAGAA
*HCF-1*siRNA	AAGGAGCTCATCGTGGTGTTT
*OGT*siRNA	CACAAUCCUGAUAAAUUU

Supplementary Table 2: PCR Primers Used in This study

Name	Sequences (5' to 3')
**RT-qPCR**	
KLF5-F (human)	ACACCAGACCGCAGCTCCA
KLF5-R (human)	TCCATTGCTGCTGTCTGATTTGTAG
Actin-F (human)	CAGGCTGCCTACCAACTCAT
Actin-R (human)	GTTCCCGAACCATCTCATTC
p27-F	AGACGGGGTTAGCGGAGCAA
p27-R	TCTTGGGCGTCTGCTCCACA
p27-F (for CHIP)	GCCGCGCTCGCCAGCCTCGG
p27-R (for CHIP)	GAGTCGCAGAGCCGTGAGCA
KLF5-F (mouse)	ACTACGGGCGAGAAGC
KLF5-R (mouse)	CCCGTGCTTCCTGTAGTG
Actin-F (mouse)	TTGCTGACAGGATGCAGAAG
Actin-R (mouse)	TGATCCACATCTGCTGGAAG
**pBABE and pGEX-6p-1 cloning**	
BAP1-F	GATCGGATCCATGAATAAGGGCTGGCTGGAG
BAP1-R	GATCGTCGACTCACTGGCGCTTGGCCTTGT
BAP1C91S-F	ACCAGCTGATACCCAACTCTTCTGCAACTCATGCCTTG CTGAGCGTGCTC
BAP1C91S-R	GAGCACGCTCAGCAAGGCATGAGTTGCAGAAGAGTTG GGTATCAGCTGGT
**pEBG cloning**	
BAP1(1-729)-F	GATCGGATCCATGAATAAGGGCTGGCTGGAG
BAP1(1-729)-R	AACGCGGCCGCTCACTGGCGCTTGGCCTTGT
BAP1(1-240)-R	GATCGCGGCCGCTCACTTGATCCTGCGGTCGGGCA
BAP1(241-596)-F	GATCGGATCCTATGAGGCCAGGCTGCATGTG
BAP1(241-596)-R	AACGCGGCCGCTCAGCTGGACCCCTGGCTGCCTT
BAP1(597-729)-F	GATCGGATCCAGCCCAGTGGAGAAGGAGGTC
**pcDNA3 cloning**	
KLF5-F	GATCGGATCCGTGCCCATGGCTACAAGGGTGCTGAGC
KLF5-R	GATCCTCGAGTCACTTGTCATCGTCATCCTTGTAATCGA TGTCATGATCTTTATAATCACCGTCATGGTCTTTGTAGT CGAATTCGTTCTGGTGCCTCTTC
KLF5(1-200)-F	GATCGGATCCATGGCTACAAGGGTGCTGAG
KLF5(1-200)-R	GATCGAATTCAGCTGCGGTCTGGTGTGAGC
KLF5(201-372)-F	GATCGGATCCATGCCAGAGGTGAACAATATTTTC
KLF5(201-372)-R	GATCGAATTCGATGCGTCGTTTCTCCAAATC
KLF5(373-457)-F	GATCGGATCCATGCACTACTGCGATTACCCTGG
KLF5(373-457)-R	GATCGAATTCGTTCTGGTGCCTCTTCATATG
**pLenti6 cloning**	
KLF5-3×Flag-F	GATCGGATCCATGGCTACAAGGGTGCTGAG
KLF5-3×Flag-R	GATCCTCGAGTCACTTGTCATCGTCATCCTTGTAATCGA

	TGTCATGATCTTTATAATCACCGTCATGGTCTTTGTAGT<br>CGAATTCGTTCTGGTGCCTCTTC
